# Convergent Patterns of Karyotype Evolution Underlying Karyotype Uniformity in Conifers

**DOI:** 10.1002/advs.202411098

**Published:** 2024-12-25

**Authors:** Ren‐Gang Zhang, Hui Liu, Hong‐Yun Shang, Heng Shu, De‐Tuan Liu, Hao Yang, Kai‐Hua Jia, Xiao‐Quan Wang, Wei‐Bang Sun, Wei Zhao, Yongpeng Ma

**Affiliations:** ^1^ Yunnan Key Laboratory for Integrative Conservation of Plant Species with Extremely Small Populations / State Key Laboratory of Plant Diversity and Specialty Crops Kunming Institute of Botany Chinese Academy of Sciences Kunming 650201 China; ^2^ University of Chinese Academy of Sciences Beijing 101408 China; ^3^ Department of Ecology and Environmental Science, Umeå Plant Science Cente Umeå University Umeå SE‐901 87 Sweden; ^4^ Institute of Crop Germplasm Resources Shandong Academy of Agricultural Sciences Ji'nan 250100 China; ^5^ State Key Laboratory of Plant Diversity and Specialty Crops and Key Laboratory of Systematic and Evolutionary Botany Institute of Botany Chinese Academy of Sciences Beijing 100093 China

**Keywords:** conifers, descending dysploidy, karyotype evolution, polyploidy, proto‐gymnosperm karyotype, reciprocal translocations, synteny analysis

## Abstract

Karyotype diversity plays an important role in speciation and diversification. However, gymnosperms, particularly conifers, exhibit remarkable karyotype uniformity. To explore the evolutionary processes shaping karyotypes in gymnosperms, the karyotype evolutionary history is reconstructed through comparative genomic analyses. Synteny analysis confirms the absence of ancient polyploidy in conifers and its rarity across the gymnosperms as a whole. Further analysis reveals convergent patterns of reciprocal translocations between nonhomologous chromosomes in conifer genomes. Centromeric‐centromeric reciprocal translocations (CRTs) have been identified as the primary mechanism of karyotype evolution in conifers, while telomeric‐centromeric reciprocal translocations (TRTs) significantly contributed to descending dysploidy within Cupressales. A graph‐based method is utilized to infer the detailed evolutionary pathways from the proto‐gymnosperm karyotype (*n* = 12) to modern conifer karyotypes (*n* = 11–12). In conclusion, the scarcity of both polyploidy and dysploidy contributes to the karyotype uniformity of gymnosperms and potentially also to their lower species richness compared to angiosperms. However, the pervasive CRTs and occasional TRTs underlie this “apparent uniformity”, supporting the “karyotype orthoselection” hypothesis. This study provides new insights into the mechanisms maintaining karyotype uniformity in conifers and the role of karyotype evolution in their diversification.

## Graphical abstract

The remarkable karyotype uniformity observed in gymnosperms has long posed an evolutionary puzzle. This study proposes a model of karyotype evolution in gymnosperms, highlighting the rarity of both polyploidy and dysploidy as key factors underlying this uniformity. The findings demonstrate that conifer karyotypes evolve primarily through centromeric–centromeric reciprocal translocations (CRTs), with telomeric–centromeric reciprocal translocations (TRTs) playing a crucial role in descending dysploidy within the Cupressales.

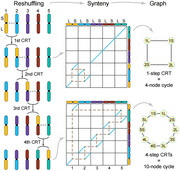

## 1 Introduction

The karyotype describes the chromosomal characteristics and reflects the structural and functional organization of the nuclear genome in eukaryotes. Karyotype constancy is crucial for the accurate transmission of genetic information across generations.^[^ ^1^, ^2^ ^]^ Nonetheless, karyotype variations can drive evolutionary change, and generally fall into two major categories: numerical and structural changes.^[^ ^1^, ^3^ ^]^ Numerical changes include dysploidy, which refers to changes in individual chromosome number, and polyploidy, involving whole genome duplication (WGD). Structural changes encompass reciprocal translocations (RTs), centromere repositioning, and other variations such as inversions, insertions, and deletions.^[^ ^4^ ^]^ These karyotype variations can trigger the emergence of innovative genetic resources and reproductive barriers, potentially leading to speciation and diversification.^[^ ^5^, ^6^, ^7^, ^8^, ^9^ ^]^ In fungi, experimental studies on karyotype engineering have demonstrated that both numerical and structural chromosome variations can directly induce strong reproductive isolation.^[^ ^10^, ^11^ ^]^ In angiosperms, where polyploidy and dysploidy are pervasive,^[^ ^7^ ^]^ a significant correlation between karyotype diversity and species richness, as well as diversification rates, suggests that karyotype evolution has likely played an important role in promoting speciation.^[^ ^8^ ^]^

In striking contrast to the angiosperms, major gymnosperm groups, particularly conifers, exhibit remarkable karyotype uniformity, as highlighted by cytogenetic studies (see reviews^[^ ^12^, ^13^ ^]^). For example, nearly all members of the Pinaceae and Cupressaceae, two predominant conifer families, have stable chromosome numbers of 2*n* = 24 and 2*n* = 22, respectively.^[^ ^12^, ^13^, ^14^ ^]^ Two main hypotheses have been proposed to explain this uniformity. The “karyotype orthoselection” hypothesis suggests that karyotype uniformity is maintained through characteristic structural mutations, whereas the “karyotype conservation” hypothesis posits that similar karyotypes are preserved across taxa due to a lack of structural mutations.^[^ ^12^ ^]^ Additionally, polyploidy is exceedingly rare in extant gymnosperms,^[^ ^12^, ^15^ ^]^ which may also contribute to this uniformity, even though WGD events have been reported in the ancestors of certain gymnosperm groups, such as the Pinaceae and Cupressaceae (see reviews^[^ ^15^, ^16^ ^]^). Comparative studies have identified numerous large‐scale inter‐chromosomal rearrangements across several conifer genomes,^[^ ^17^, ^18^, ^19^, ^20^, ^21^, ^22^ ^]^ lending tentative support for the “karyotype orthoselection” hypothesis. However, a comprehensive, in‐depth analysis across all major gymnosperm groups remains lacking, leaving the evolutionary processes that have shaped gymnosperm karyotypes largely unexplored.

Although gymnosperms usually have extraordinarily large and complex genomes, rapid advancements in genome sequencing techniques have now enabled the production of high‐quality genome assemblies for all major gymnosperm clades,^[^ ^16^ ^]^ providing an unprecedented opportunity to address the longstanding questions through comparative genomic analyses. In this study, we analyzed 16 chromosome‐level genome assemblies from gymnosperms to reconstruct the evolutionary trajectories of karyotypes within major groups. Our findings reveal convergent patterns of karyotype evolution across conifer genomes, shedding light on the mechanisms underlying karyotype uniformity in gymnosperms.

## 2 Results

### 2.1 Whole Genome Duplications in Gymnosperms

**Figure 1:**
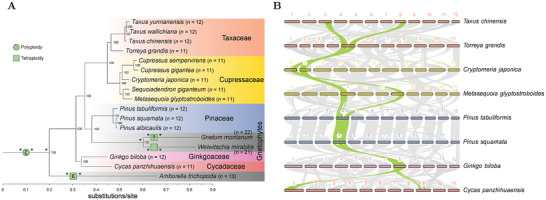
Rare WGD events in gymnosperm genomes. A) Phylogenetic tree showing inferred relationships and WGD events in the studied gymnosperm species. The tree is rooted using *Alsophila spinulosa* (fern) and *Physcomitrium patens* (moss) (not shown). Values on the nodes represent bootstrap values. *n* indicates the haploid chromosome number. Note that the evidence supporting the tetraploidy hypothesis in the ancestry of *Gnetum montanum* is quite weak (indicated by “?”), and further validation is needed. Asterisks (*) indicate inner nodes where karyotype reconstruction was not possible due to extremely fragmented synteny. B) One‐to‐one, arm‐scale synteny patterns among genomes of conifers, ginkgo, and cycad, demonstrating the absence of WGD across these genomes. One typical case of arm‐scale syntenic blocks is highlighted in green. Comparisons with other conifer genomes in panel A also reveal the similar 1:1 synteny patterns (not shown). For more evidence of WGD inference, refer to Figures S1–S3 (Supporting Information).

To investigate karyotype evolution in gymnosperms, we sampled 16 species with chromosome‐level genome assemblies, representing all major clades within the group (**Figure** **1**A; Table S1, Supporting Information). The *Alsophila spinulosa* (fern), *Physcomitrium patens* (moss) and the most basal angiosperm, *Amborella trichopoda*, were included as outgroups. Phylogenomic analyses based on 836 single‐copy genes inferred a tree topology (Figure 1A) consistent with previous studies.^[^ ^23^, ^24^, ^25^ ^]^ Given the significant impact of WGDs on karyotype evolution, we subsequently conducted orthologous synteny analysis^[^ ^26^ ^]^ to investigate the presence of WGDs in gymnosperms. Pairwise comparisons among *Ginkgo biloba* (Ginkgoaceae), *Cycas panzhihuaensis* (Cycadaceae), Pinaceae (e.g., *Pinus squamata* and *P. tabuliformis*), Cupressaceae (e.g., *Metasequoia glyptostroboides* and *Cryptomeria japonica*) and Taxaceae (e.g., *Taxus chinensis* and *Torreya grandis*) showed a consistent 1:1 syntenic depth ratio (Figure 1B; Figure S1, Supporting Information). These arm‐scale, one‐to‐one syntenic patterns strongly suggest the absence of independent WGD events in these families (Figure 1). This finding therefore rejects the hypothesis that the separate ancestries of Pinaceae and Cupressaceae have undergone independent WGD events, as previously suggested.^[^ ^20^, ^27^, ^28^ ^]^ If these two independent WGD events had occurred, we would expect a 2:1 syntenic depth ratio between Pinaceae/Cupressaceae and *G. biloba*/*C. panzhihuaensis*, and a 2:2 ratio between Pinaceae and Cupressaceae.^[^ ^26^ ^]^ Previous inferences of ancient WGD in these two families relied heavily on evidence from gene trees and/or distributions of synonymous substitution rates (*Ks*).^[^ ^20^, ^25^, ^27^, ^28^ ^]^ In such cases, misinterpretation is difficult to avoid due to the lack of sufficient consideration of inter‐genomic synteny, which may result in confusion with other types of gene duplications,^[^ ^26^ ^]^ such as transposed or segmental duplicates, that likely occurred in the ancestries of the Pinaceae and Cupressaceae.

In the genomes of the gnetophytes (*Gnetum montanum* and *Welwitschia mirabilis*), the chromosome numbers (*n* = 21 or 22) are approximately double those of other gymnosperms (*n* = 11 or 12) (Figure 1A), hinting the possible occurrence of WGD events in gnetophytes. Consistently, the *W. mirabilis* genome exhibited a 2:1 synteny depth ratio relative to the *G. biloba* genome, with ≈200 sliding gene windows (window size = 50 and step = 10) showing such synteny pattern (Figure S2A, Supporting Information). Furthermore, ≈700 gene windows within the *W. mirabilis* genome exhibit 1:1 synteny, with a clear *K*s peak (≈1.0) for the paralogous syntenic genes (Figure S2D, Supporting Information). These patterns indicates a lineage‐specific ancient tetraploidy event in *W. mirabilis* (Figure 1A) and also corroborates the previous inference based on various lines of evidence.^[^ ^25^, ^27^, ^29^, ^30^ ^]^ However, clear synteny patterns were not observed in the *G. montanum* genome, likely due to massive chromosomal rearrangements and gene fractionation. Nevertheless, a few 2:1 synteny patterns relative to *G. biloba* (Figure S2B, Supporting Information) supported the possibility of an ancient tetraploidy event in *G. montanum* (Figure 1A). Given the uncertainty caused by the extremely fragmented synteny (Figure S2, Supporting Information), additional high‐quality gnetophyte genomes are still required to confirm these WGD events. Meanwhile, the 2:1 syntenic pattern between *A. trichopoda* and *G. biloba* suggested an ancient tetraploidy event at *Ks* ≈ 1.75 in *A. trichopoda* (Figure 1A; Figure S2C,F, Supporting Information), corresponding to the previously identified angiosperm‐common ε‐WGD event.^[^ ^31^ ^]^ However, this 2:1 synteny pattern contradicts the gymnosperm‐common ω‐WGD hypothesis,^[^ ^24^, ^25^ ^]^ which predicts a 2:2 (or 1:2) synteny between *A. trichopoda* and *G. biloba*. Instead, our synteny analysis supports the seed pant common ζ‐WGD,^[^ ^31^ ^]^ which occurred at *Ks* ≈ 0.8 and left numerous syntenic relics in *G. biloba*, although the ζ‐WGD‐derived syntenic blocks were nearly undetectable in the other genomes (Figure S3, Supporting Information). Overall, in striking contrast to angiosperms, WGDs are rare not only in modern gymnosperms^[^ ^12^, ^15^, ^32^ ^]^ but also in their ancestral lineages (Figure 1A), resulting in negligible effects on karyotype evolution in gymnosperms (excluding gnetophytes), and contributing to the karyotype uniformity in groups such as conifers.

As the subsequent karyotype evolution analyses rely on conserved synteny,^[^ ^33^, ^34^ ^]^ the genomes of the two gnetophytes and *A. trichopoda* were excluded. Massive chromosomal rearrangements, likely resulting from post‐polyploidization diploidization processes, caused extremely fragmented synteny within these genomes (Figure S2, Supporting Information), rendering the evolutionary paths of their karyotype changes untraceable.

### 2.2 Centromere Identification and Karyotype Symmetry in Gymnosperms

The putative centromere positions of nine representative gymnosperms species (*G. biloba*, *C. panzhihuaensis*, *P. squamata, P. tabuliformis*, *M. glyptostroboides*, *C. japonica*, *Cupressus gigantea*, *T. chinensis*, and *T. grandis*) were identified using Hi‐C interaction heatmaps constructed in this study (Figure S4, Supporting Information) and from previous studies.^[^ ^20^, ^35^, ^36^ ^]^ These Hi‐C contact maps revealed that all these genomes exhibited Rabl configurations, characterized by intense interactions between chromosome arms (X‐shape) and between telomeres, as well as between centromeres.^[^ ^37^, ^38^ ^]^ Based on approximate centromere positions identified by the Hi‐C signals, nearly all chromosomes in the studied conifer genomes are metacentric, with arm ratios <1.68 (Table S2, Supporting Information). However, exceptions include two telocentric chromosomes (chr10 and chr12) in *T. chinensis* and one subtelocentric chromosome (chr9) in *C. gigantea* (Table S2, Supporting Information). In contrast, increased karyotype asymmetry was observed in the Ginkgoaceae and Cycadaceae, with six subtelocentric chromosomes identified in *G. biloba* and seven subtelocentric/telocentric chromosomes in *C. panzhihuaensis* (Table S2, Supporting Information). These genome‐based findings generally align with previous cytogenetic studies.^[^ ^12^, ^13^, ^39^, ^40^, ^41^, ^42^ ^]^

We further analyzed the repeat sequences within the pericentromeric regions. The genomes of *P. squamata, C. japonica*, and *T. grandis*, assembled using PacBio High‐Fidelity reads, enabled the accurate annotation of long centromeric satellite arrays. Long, putative centromeric satellite arrays were identified in the pericentromeric regions of these genomes, with repeat unit lengths of 148, 148, and 102 bp, respectively, and without sequence identity between these motifs (Figure S5, Supporting Information). For genomes assembled using long noisy reads (PacBio CLR or Nanopore), centromeric satellite arrays with long repeat units (> 50 bp) were limited (Figure S5, Supporting Information), likely due to the collapsed assembly of the excessively long tandem repeated sequences. Nevertheless, putative centromeric satellite arrays with a 7 bp repeat unit were observed in the pericentromeric regions of most of these genomes, with the exception of *P. tabuliformis* (Figure S5, Supporting Information). Notably, telomere‐like 7 bp satellite repeat sequences (TTTAGGG or TTTAGGG‐like) were enriched in the pericentromeric regions of *T. chinensis* and *P. squamata*, consistent with previous cytogenetic studies for the two genera.^[^ ^43^, ^44^ ^]^ The concurrences of satellite arrays support the high confidence of the approximate centromere regions characterized by Hi‐C signals. In addition to tandem repeats, transposable elements (TEs) were also highly abundant in these regions (Figure S6, Supporting Information). The enrichment of these repetitive elements led to extensive sequence similarities across centromeric regions within each species, as revealed by all‐versus‐all alignments (Figure S7, Supporting Information).

### 2.3 Karyotype Evolution in Gymnosperms

**Figure 2:**
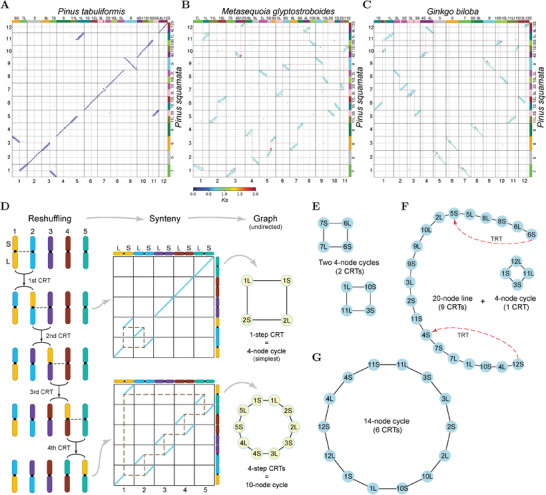
Centromere‐aware synteny analyses of gymnosperm genomes suggesting a CRT model for the arm‐scale rearrangements. A–C) Typical arm‐reshuffling patterns indicated by synteny (dot plots) between *Pinus squamata* and *P. tabuliformis* (A), *Metasequoia glyptostroboides* (B), and *Ginkgo biloba* (C). Syntenic blocks were colored by median *K*s values. Numbers along the bottom and left axes of the dot plots represent chromosomes in modern genomes. Colored panels and numbered labels along the top and right axes indicate the mapped chromosomes/arms of the proto‐gymnosperm karyotype (PGK). Dashed gray lines within chromosomes denote centromere positions. Reshuffled arms are highlighted by colored shadows, and breakpoints are linked by red dashed polygons. Centromere repositioning is indicated by blue arrows. D) A model involving successive CRTs to explain the arm‐reshuffling patterns. Note that the multiple CRTs are unordered. E–G) Typical graphs showing the arm‐reshuffling patterns between *P. squamata* and *P. tabuliformis* (E), *M. glyptostroboides* (F), and *G. biloba* (G). Nodes in these graphs are unified by using the arm numbers from the PGK, corresponding to the arm numbers along on the top and right axes of the dot plots (A–C). Edges (black lines) indicate links by breakpoints. CRT: centromeric–centromeric reciprocal translocation; TRT: telomeric–centromeric reciprocal translocation.

Based on synteny analyses with the known centromere positions, we found convergent patterns of reciprocal translocations (RTs) between non‐homologous chromosomes in conifer genomes (**Figure** **2**). For example, two arm‐scale RTs were observed between two pine species (*P. squamata* and *P. tabuliformis*), involving exchanges between chromosome 1 (chr1) and chr3, and between chr5 and chr11 (Figure 2A). Remarkably, when comparing *P. squamata* and *M. glyptostroboides*, all chromosomal arms appeared to have been reshuffled via arm‐scale RTs (Figure 2B). The breakpoints of all these RTs were located within or near the centromeres, leading us to refer to them as centromeric–centromeric reciprocal translocations (CRTs). When comparing *P. squamata* with *G. biloba*, the arms of seven chromosomes were reshuffled, while five chromosomes (chr1, chr2, chr3, chr4, and chr9 in *P. squamata*) retained intact (Figure 2C). These reshuffling patterns likely resulted from successive CRTs, which can be simplified using an arm‐reshuffling graph, where chromosomal arms are represented as nodes and centromeric breakpoints as edges (Figure 2D). In these empirical data, these graphs are generally cyclic, except for a linear component observed between *P. squamata* and *M. glyptostroboides* (Figure 2E–G). During successive CRTs, a chromosomal arm can be transferred and recombined with another arm through multiple CRT events. In the process, CRTs may recur at the same centromere region, with breakpoints shifting due to centromere repositioning or breaks occurring at varying positions around the centromere (Figure 2B,C). For example, centromere repositioning was observed in 10 of the 12 chromosomes when comparing *P. squamata* and *G. biloba* (Figure 2C).

**Figure 3:**
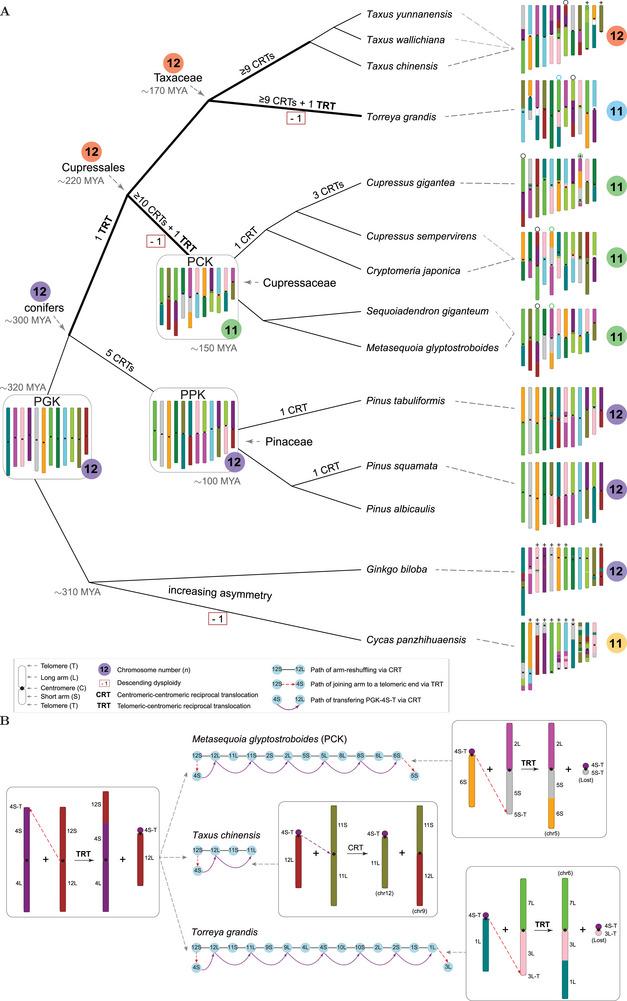
Karyotype evolution in gymnosperms. A) Summary of karyotype evolution in major clades of gymnosperms (excluding gnetophytes). Ancestral karyotypes: PGK, proto‐gymnosperm karyotype; PPK, proto‐Pinaceae karyotype; PCK, proto‐Cupressaceae karyotype. Large‐scale inter‐chromosomal rearrangements: CRT, reciprocal translocation with both breakpoints occurring within or near centromeres; TRT, reciprocal translocation with one breakpoint occurring near a telomere and the other within or near a centromere. Bold branches in the tree indicate possible saturation of CRTs. Numbers in colored circles indicate the haploid chromosome numbers (*n*), with the same colors indicating similar karyotypes. Estimated ages at ancestral nodes were compiled from previous studies.^[^ ^24^, ^45^ ^]^ MYA, million years ago. Numbers in boxes (−1) indicate a reduction in chromosome number (descending dysploidy). Symbol “°” indicates chromosomes recombined by TRTs in modern Cupressales genomes. Telocentric or subtelocentric chromosomes (arm ratio > 3.0) in modern genomes are marked with “+”, showing increasing asymmetry in some genomes. For more details on karyotype evolutionary trajectories, refer to Figures S8–S13 (Supporting Information). B) Different fates of the telocentric chromosome (PGK‐12L + PGK‐4S‐T) in *Metasequoia glyptostroboides*, *Taxus chinensis*, and *Torreya grandis*. Colored circles with arm numbers of PGK and black lines indicate arm‐reshuffling graphs via CRTs. In the graphs, red dashed arrows indicate TRTs joining an arm to the telomeric end of another chromosome, and purple arrows show the transfer of the short arm (PGK‐4S‐T) of the telocentric chromosome via CRTs. Only the first and last events are illustrated using a schematic diagram of chromosomal rearrangements. Schematic diagrams of the other intermediate CRTs for the transfer of PGK‐4S‐T can be found in Figure S10G–H (Supporting Information). In the diagrams, chromosome identification numbers in brackets correspond to those in extant genomes.

The extensive CRT‐driven reshuffling patterns observed across conifer genomes have provided valuable insights into gymnosperm karyotype evolution. To further interpret these patterns, we reconstructed the proto‐gymnosperm karyotype (PGK) to trace the evolutionary processes shaped by CRTs and to explore the mechanisms underlying dysploidy in gymnosperms. Determining the ancestral state of CRT events is challenging due to the absence of an appropriate outgroup. To address this, we tentatively designated the chromosomes of *G. biloba* as ancestral for CRT breakpoints, based on the synteny patterns with *C. panzhihuaensis* (Figure S1A, Supporting Information). However, this designation remains provisional without unambiguous confirmation from additional outgroups (see Methods for details). Moreover, since most centromere positions of *G. biloba* are not conserved with those in conifers, we incorporated the chromosomal arms from *P. squamata* to reconstruct the ancestral centromere positions (see Methods for explanations). Consequently, the PGK integrates arms from *P. squamata*, recombined in a pattern analogous to *G. biloba*. The PGK comprises 12 metacentric chromosomes (PGK1 to PGK12) and 24 arms, with each proto‐chromosome consisting of a long arm (L), a short arm (S), a centromere (C) and two telomeres (T) (**Figure** **3**A). We subsequently mapped the PGK to the extant genomes and constructed arm‐reshuffling graphs to illustrate the minimized evolutionary path through successive CRTs (Figure 3; Figures S8–S12, Supporting Information), thereby simplifying the complex karyotype evolutionary history and highlighting key chromosomal changes.

Based on the PGK, five CRTs led to the formation of the most recent common ancestor (MRCA) of the three studied pine genomes (PPK), whose karyotype is analogous to that of *P. albicaulis* (Figure 3A; Figure S8, Supporting Information). Following this, species‐specific CRTs occurred: one between chr1 and chr3 in *P. tabuliformis* and another between chr5 and chr11 in *P. squamata* (Figure S9, Supporting Information). Despite these species‐specific karyotype differences, the overall karyotype remains highly conserved within the *Pinus* lineage, aligning to the overall karyotypic uniformity observed across the Pinaceae family.^[^ ^12^, ^13^ ^]^

**Figure 4:**
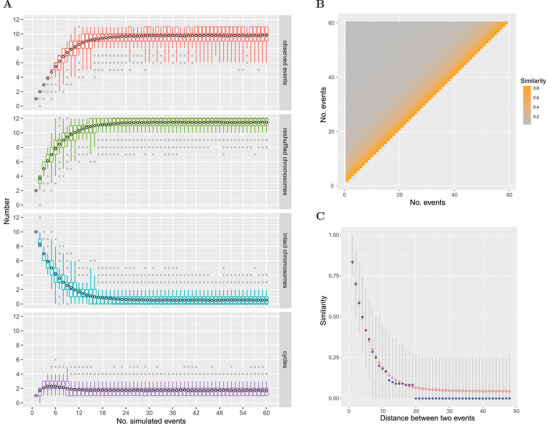
Simulation of successive CRTs. A) Boxplots showing the numbers of observed CRT events, reshuffled chromosomes, intact chromosomes, and circles in arm‐reshuffling graphs across 1 to 60 simulated successive CRT events. Black rhombuses represent the mean values. B,C) Similarity between reshuffled chromosomes from two events. In panel C, blue points represent median values, and red rhombuses represent mean values. Gray lines indicate 95% confidence intervals.

In striking contrast to the limited number of CRTs in pines, numerous CRTs occurred across Cupressales genomes, with reshuffling and recombination observed in all chromosomal arms, except for chr5 in *T. grandis* (Figure 3; Figure S10A–C, Supporting Information). Simulation analyses (**Figure** **4**) suggest that chromosomal reshuffling within Cupressales genomes is likely saturated due to the high frequency of CRTs. Some of these CRTs may have led to back recombination events, complicating the reconstruction of precise evolutionary paths from the PGK. Nevertheless, we proposed a putative karyotype evolution model using the maximum‐parsimony evolutionary paths (Figure S10D–F, Supporting Information). Interestingly, these paths indicated that the studied groups of the Cupressales (Cupressaceae, *Taxus*, and *Torreya*) likely shared a recombination event that fused the short arm of PGK12 (PGK‐12S) with PGK‐4S via an RT event (Figure 3B; Figures S10 and S11, Supporting Information). One breakpoint of the RT occurred near or within the telomeric region of PGK‐4S, while the other was located within or near the centromeric region of PGK12. This resulted in the formation of a telocentric chromosome comprising a long arm (PGK‐12L) and a particularly short arm derived from the telomeric end of PGK‐4S (hereafter, PGK‐4S‐T) (Figure 3B). We designate this type of RT as a telomeric–centromeric reciprocal translocation (TRT). Following this common TRT event, the fates of the resulting telocentric chromosome (PGK‐12L + PGK‐4S‐T) diverged among the three different lineages (Figure 3B). In the MRCA of the Cupressaceae (PCK), PGK‐4S‐T underwent at least four translocations before eventually recombining with PGK‐6S via CRTs (Figure 3B; Figure S10G, Supporting Information). Subsequently, PGK‐6S fused with PGK‐5S, while PGK‐4S‐T combined with PGK‐5S‐T via another TRT, forming a chromosome with two particularly short arms (PGK‐4S‐T + PGK‐5S‐T) (Figure 3B). This chromosome was eventually lost, reducing the chromosome number from *n* = 12 to *n* = 11, and resulting in descending dysploidy in the Cupressaceae. Similarly, in *Torreya*, PGK‐4S‐T underwent at least five translocations before recombining with PGK‐1L, finally fusing with the PGK‐3L‐T via TRT (Figure 3B; Figure S10H, Supporting Information), leading to the loss of this particularly short chromosome (PGK‐4S‐T + PGK‐3L‐T) and a reduction in chromosome number to *n* = 11. In *Taxus*, however, PGK‐4S‐T recombined with PGK‐11L, resulting in the preservation of a short, telocentric chromosome (PGK‐4S‐T + PGK‐11L) (Figure 3B). This chromosome persists present today (chr12 of *T. chinensis*, Figure S4, Supporting Information) and has also been observed cytogenetically.^[^ ^39^ ^]^ Within the Cupressaceae, CRTs only occurred with a very limited number (0–3) (Figure 3A; Figure S12, Supporting Information), consistent with the overall uniformity of karyotypes within this family.^[^ ^12^, ^13^ ^]^ The karyotypes of the three studied *Taxus* species remained conserved, showing no species‐specific CRTs (Figure 3A; Figure S13, Supporting Information). Notably, both *C. gigantea* and *Taxus* spp. exhibited a telocentric/subtelocentric chromosome (chr9 of *C. gigantea* and chr10 of *T. chinensis*), likely derived from pericentric inversions (Figures S10–S13, Supporting Information). Interestingly, these chromosomes with (sub‐)telocentric morphology were not observed in previous karyomorphological studies.^[^ ^39^, ^46^ ^]^

In *G. biloba*, the chromosome number remains at *n* = 12, but most centromere positions have shifted to one side of the chromosomes (Figure 3A; Figure S14A, Supporting Information), leading to increased karyotype asymmetry, with most chromosomes now being subtelocentric or submetacentric (see also cytologenetic karyotype^[^ ^40^ ^]^). The centromere shifts or repositioning were likely mediated by gradual pericentric inversions,^[^ ^4^ ^]^ as multiple small‐scale inversions were observed between the centromere positions of homoeologous chromosomes in PGK and *G. biloba* (Figure 2C; Figure S14A, Supporting Information). In *C. panzhihuaensis*, the chromosome number has reduced to *n* = 11, exhibiting complex rearrangements and different patterns compared to conifers. One proto‐chromosome (PGK1) appears to have undergone centric fission, while two others (PGK2 and PGK5) were fragmented and subsequently joined with other chromosomes, possibly via unequal RTs. The majority of each of the 11 chromosomes corresponds to a single proto‐chromosome (Figure S14B, Supporting Information), contrasting sharply with the CRTs model commonly observed in conifer genomes. Most chromosomes of *C. panzhihuaensis* are telocentric or subtelocentric (Figure S4, Supporting Information; for karyotype asymmetry in *Cycas* also see review^[^ ^42^ ^]^) contrasting with the predominance of metacentric chromosomes in conifers. These findings highlight the uniqueness of karyotype evolution in cycads. Additional cycad genomes may provide crucial insight into the specific paths of dysploidy within this lineage. Nevertheless, the chromosome numbers (*n* = 11–12) in these gymnosperms reflect an ancient diploid origin (no lineage‐specific WGDs), suggesting remarkable uniformity of chromosome numbers in these lineages (Figure 3A). However, as our study reveals, this “apparent uniformity” is underpinned by significant chromosomal rearrangements.

## 3 Discussion

We identified frequent, arm‐scale RTs across conifer genomes, primarily characterized by CRTs. Additionally, we discovered three TRTs within the Cupressales (Figure 3). CRTs result in the reshuffling of arms from nonhomologous chromosomes, whereas TRTs involve the fusion of an arm from its centromeric end to the telomeric end of another chromosome. Both types of RTs showed convergent patterns that occurred independently in different lineages but within similar genomic regions associated with centromeres and/or telomeres. CRTs are likely facilitated by two main factors. First, Rabl configurations, which are prevalent in conifer genomes, bring centromeres into close physical proximity, promoting frequent interactions and translocations between nonhomologous (peri‐)centromeres (Figure S4, Supporting Information). This configuration has also been observed across diverse organisms, including animals, yeast, and plants with large genomes such as barley, oat, rye, and wheat.^[^ ^37^, ^47^ ^]^ Centric translocations resembling the CRTs in conifers have been reported in the wheat–rye crosses.^[^ ^48^ ^]^ Close physical proximity resulting from chromosomal configurations also underlies other types of translocations, such as end‐to‐end fusions in angiosperms.^[^ ^49^ ^]^ Second, the abundance of repetitive elements, including centromeric satellites and TEs, in the (peri‐)centromere regions of conifer genomes (Figures S5–S7, Supporting Information) likely facilitates non‐allelic homologous recombination‐mediated CRTs.^[^ ^4^ ^]^ Notably, recurrent CRTs can occur at varying positions around a given centromere when mediated by different repetitive elements, despite the primary constraints conferred by Rabl configurations. Similar phenomena of constricted yet varied breakpoint positions have been reported in the wheat–rye translocations.^[^ ^48^ ^]^ This scenario explains the small‐scale breakpoint shifts observed in this study (e.g., Figure 2B,C). Consequently, identifying the specific repeat sequences mediating CRTs is challenging due to the extreme abundance of repetitive sequences around the centromere regions (Figures S5–S7, Supporting Information) and the rapid evolution of centromeres.^[^ ^50^ ^]^

In contrast to CRTs, the factors promoting TRTs remain unclear. While Rabl configurations facilitate CRTs by bringing centromeres into proximity, they spatially separate centromeres and telomeres, reducing the likelihood of centromere–telomere interactions, which are likely required for TRTs. As a result, TRTs may arise under rare, accidental conditions, possibly explaining their much lower incidence compared to CRTs within Cupressales. Alternatively, TRTs could occur during meiosis, where bouquet formation of telomeres may facilitate centromere–telomere interactions,^[^ ^49^ ^]^ and create accidental opportunities for such translocations. In addition, an alternative mechanism for the common TRT event in Cupressales (Figure 3B) likely involves a two‐step process: first, a centric fission event that produces two telocentric chromosomes (PGK12S + T and PGK12L + T) stabilized by *de novo* addition of telomeres, followed by the fusion of one of these chromosomes (PGK12S + T) with another metacentric chromosome (PGK4). However, this two‐step scenario appears less likely than our one‐step model, which does not require the capture or *de novo* synthesis of telomeres (Figure 3B). In fact, centric fission is quite rare in plants.^[^ ^4^, ^51^ ^]^

When a TRT produces a particularly short chromosome with few essential genes, that chromosome may be lost without significantly affecting the organism, giving rise to the descending dysploidy seen in the Cupressales (Figure 3B). Such a TRT can be considered as a form of end‐to‐end fusion, and appears to be a primary mechanism underlying descending dysploidy in conifers, contrasting with the more diverse mechanisms observed in angiosperms.^[^ ^4^, ^49^, ^51^ ^]^ In angiosperms, descending dysploidy can occur through various processes, including multiple types of end‐to‐end fusions and nested chromosome fusions.^[^ ^34^, ^49^, ^52^ ^]^ However, due to the upper size limit of eukaryotic chromosomes set by the spindle axis at telophase,^[^ ^49^, ^53^ ^]^ the ultra‐long chromosomes in gymnosperms may inhibit other mechanisms of descending dysploidy, such as an end‐to‐end fusion of two long, metacentric chromosomes: a process common in angiosperms and facilitated by the close proximity of telomeres in Rabl configurations.^[^ ^49^ ^]^ The paucity of these mechanisms may contribute to the uniformity of chromosome numbers in most conifers, particularly in the Pinaceae and Cupressaceae, despite the extensive reshuffling via CRTs observed in this study (Figure 3A). Therefore, our findings support the “karyotype orthoselection” hypothesis, which posits that karyotype uniformity is maintained through the occurrence of “characteristic structural mutations”,^[^ ^12^ ^]^ such as the frequent CRTs (Figure 3A). However, the maintenance of karyotype uniformity may not necessarily be driven by selection, but instead primarily constrained by inherent factors, such as the chromosomal configurations discussed above.

Both CRTs and TRTs may drive or reinforce speciation in conifers, as these arm‐scale RTs can lead to reproductive barriers. In angiosperms, polyploidy and dysploidy are frequent, leading to a diversity of chromosome numbers (a proxy for karyotypic diversity), which has been linked to higher species richness and increased diversification rates.^[^ ^7^, ^8^ ^]^ In contrast, polyploidy and dysploidy are rare in gymnosperms (with the exception of *Ephedra* and *Juniperus*), leading to uniform chromosome numbers (except in the Podocarpaceae), which may contribute to their lower species richness compared to angiosperms.^[^ ^54^ ^]^ Nevertheless, the frequent RTs in gymnosperms could enhance karyotypic diversity and potentially increase speciation rates, partially compensating for the lack of polyploidy and dysploidy. However, the constrained variability in the breakpoints of these RTs might limit the overall extent of karyotypic diversity in gymnosperms. In fact, as observed in this study, gymnosperm karyotypes, have remained much more conserved over the past 300 million years (Myr)^[^ ^23^, ^24^ ^]^ than those of the angiosperms.^[^ ^34^ ^]^ Our analysis of karyotype evolution provides valuable insights into gymnosperm diversification. With the availability of additional chromosome‐level genome assemblies and broader taxon sampling in gymnosperms, deeper insights may be revealed for the roles of karyotype evolution in driving diversification.

It is also important to acknowledge the uncertainties and limitations of this study. First, the ancestral karyotype estimation involves several empirical and subjective decisions. These decisions lack unambiguous support from outgroups and are therefore potentially biased. However, these biases primarily affect the phylogenetic placements of a few CRT events (e.g., those in the ancestry of the Pinaceae) and do not impact on the observed patterns and the overall robustness of the conclusions, due to the employment of the graph‐based method. Second, the inference of WGDs in the gnetophytes genomes, especially the putative WGD event in *G. montanum*, lacks strong evidence due to the extremely fragmented synteny, leaving alternative scenarios open to consideration. Nevertheless, we propose that the gnetophytes WGD hypothesis remains a compelling explanation for the double chromosome numbers and the highly fragmented synteny, which are likely consequences of extensive diploidization driven by ancient WGDs in gnetophytes. Future research with additional genomic data will be essential to address these uncertainties and limitations.

## 4 Conclusion

The study addresses the longstanding puzzle of the evolutionary dynamics of gymnosperm genomes, particularly their remarkable karyotype uniformity compared to that of angiosperms. Comparative genomic and synteny analyses reveal a notable absence of ancient polyploidy in conifer genomes, and conifer karyotype evolution has predominantly been driven by centromeric–centromeric reciprocal translocations (CRTs), while telomeric–centromeric reciprocal translocations (TRTs) have played a key role in descending dysploidy in the Cupressales. The rarity of both polyploidy and dysploidy events in gymnosperms leads to an overall uniformity of chromosome numbers and potentially contributes to their lower species richness relative to angiosperms. This study presents an insightful framework for understanding karyotype evolution in gymnosperms, shedding light on the evolutionary mechanisms that have shaped their genomes.

## 5 Experimental Section

### Genomic Data Collection

The genomic data for *Cryptomeria* *japonica*,^[^ ^55^ ^]^ *Cupressus* *gigantea*,^[^ ^35^ ^]^ *Cupressus* *sempervirens* (Genbank: GCA_028749045.1), *Cycas* *panzhihuaensis*,^[^ ^24^ ^]^ *Ginkgo* *biloba*,^[^ ^56^ ^]^ *Gnetum* *montanum*,^[^ ^29^ ^]^ *Metasequoia* *glyptostroboides*,^[^ ^18^ ^]^ *Pinus* *albicaulis*,^[^ ^57^ ^]^ *Pinus* *squamata*,^[^ ^36^ ^]^ *Pinus* *tabuliformis*,^[^ ^20^ ^]^ *Sequoiadendron* *giganteum*,^[^ ^58^ ^]^ *Taxus* *chinensis*,^[^ ^59^ ^]^ *Taxus* *wallichiana*,^[^ ^17^ ^]^ *Taxus* *yunnanensis*,^[^ ^21^ ^]^ *Torreya* *grandis* ^[^ ^19^ ^]^ and *Welwitschia* *mirabilis*,^[^ ^29^ ^]^ as well as the outgroups including *Amborella* *trichopoda*,^[^ ^60^ ^]^ *Alsophila* *spinulosa* ^[^ ^61^ ^]^ and *Physcomitrium* *patens*,^[^ ^62^ ^]^ were obtained from public databases or were provided by the corresponding authors. Two genomes, *C. sempervirens* and *T. wallichiana*, were annotated using MetaEuk v4^[^ ^63^ ^]^ due to the lack of gene annotations. The longest protein of each gene was selected for downstream analyses. Genome assembly and gene annotation quality were assessed with miniBUSCO^[^ ^64^ ^]^ and BUSCO v5.3.2,^[^ ^65^ ^]^ respectively, using the embryophyta_odb10 database (1614 conserved genes). Details of the quality assessments for the gymnosperm genomes are provided in Table S1 (Supporting Information).

### Centromere Identification and Characterization

Putative centromeric positions were identified using Hi‐C interaction heatmaps generated with Juicer v1.5.6,^[^ ^66^ ^]^ with Hi‐C data downloaded from public databases. In chromatin with a Rabl configuration, inter‐chromosomal signals around centromere regions have been characterized.^[^ ^67^ ^]^ Based on these signals, the approximate centromere positions were manually identified from the Hi‐C contact maps using the Juicebox software.^[^ ^68^ ^]^ For *C. gigantea* and *P. tabuliformis*, whose Hi‐C raw data are not publicly available, centromere positions were estimated from published Hi‐C contact maps.^[^ ^20^, ^35^ ^]^ For *P. squamata*, the previously identified centromere positions, primarily determined by Hi‐C interactions,^[^ ^36^ ^]^ were used directly. Chromosome types were classified based on arm ratios (long arm length/short arm length) using a modified nomenclature,^[^ ^69^, ^70^ ^]^ which is particularly practical for genomic sequences (arm ratio in square brackets): metacentric [1.00–1.67], submetacentric [1.68–3.00], subtelocentric [3.01–7.00] and telocentric [> 7.00].

To confirm the putative centromere positions, the repeat sequences were further analyzed within these regions. Sequences of 30 Mb upstream and downstream of the putative centromere positions (pericentromere regions) were extracted and tandem repeats were analyzed using TRASH v1.2.^[^ ^71^ ^]^ Transposable elements were annotated using the EDTA pipeline v1.9.9 with the parameter “–anno 1”.^[^ ^72^ ^]^ The sequences of centromere regions were then aligned using MMseqs2 v13.45111.^[^ ^73^ ^]^

### Identification of Synteny and Inference of Orthology

An all‐versus‐all BLAST search of protein sequences for each species pair was executed using DIAMOND v0.9.24.^[^ ^74^ ^]^ Orthologs were inferred utilizing OrthoFinder v2.3.1, with the parameter “‐M msa”.^[^ ^75^ ^]^ Syntenic blocks were identified using WGDI v0.6.2 with the `‐icl` option and default parameters.^[^ ^33^ ^]^ The synonymous substitution rate (*Ks*) for syntenic gene pairs was estimated using the `‐ks` option of WGDI. Orthologous syntenic blocks were further identified through the Orthology Index by integrating both orthology and synteny information.^[^ ^26^ ^]^

### Reconstruction of Species Tree

The species tree based on nuclear DNA was reconstructed employing the SOI pipeline.^[^ ^26^ ^]^ In brief, orthologs identified with OrthoFinder were extracted, and protein sequences were aligned using MAFFT v7.481.^[^ ^76^ ^]^ Codon alignments were then converted from the protein alignments with PAL2NAL v14,^[^ ^77^ ^]^ and subsequently trimmed using trimAl v1.2 with the parameter “‐gappyout”.^[^ ^78^ ^]^ A concatenated alignment of 836 single‐copy genes was generated (SOI parameter: “phylo ‐sc ‐mm 0.2 ‐concat”, allowing up to 20% missing taxa) to reconstruct a phylogenetic tree using IQ‐TREE v2.2.0.3,^[^ ^79^ ^]^ with 1000 bootstrap replicates. The resulting tree topology was identical with that inferred via the coalescent‐based method ASTRAL‐Pro v1.15.2.3.^[^ ^80^ ^]^

### Inference of Polyploidy

Polyploidy was inferred by examining orthologous synteny patterns between genomes.^[^ ^26^ ^]^ For example, the *P. squamata* genome showed a clear 1:1 orthologous syntenic depth ratio relative to the *G. biloba* genome (Figure 2C), indicating that neither genome has undergone any lineage‐specific WGD events. If *P. squamata* (or the ancestral Pinaceae) had experienced an independent WGD, the syntenic depth ratio would have been 2:1. The putative polyploidy events were further validated using the *Ks* distribution patterns of paralogous syntenic genes, as a distinct *Ks* peak is expected for a polyploidy event. This strategy was consistently applied to infer paleopolyploidy across other genomes.

### Reconstruction of Ancestral Karyotypes

Following the methodology of Sun et al,^[^ ^33^, ^34^ ^]^ the ancestral karyotypes in gymnosperms were reconstructed by identifying chromosome‐scale orthologous synteny retained intact across multiple genomes. Intra‐chromosomal rearrangements, including inversions, insertions/deletions, duplications, and translocations, were ignored. For proto‐gymnosperm karyotype (PGK) reconstruction, dot plots revealed that five of the twelve *G. biloba* chromosomes (chr5, 6, 7, 8, and 9) display chromosome‐scale synteny with *P. squamata* or *P. albicaulis*, three (chr5, 8 and 9) with *P. tabuliformis*, four (chr6, 7, 11 and 12) with *C. panzhihuaensis*, one (chr8) with *T. grandis*, and none with other studied gymnosperms genomes (Figure 2C; Figure S1, Supporting Information). Thus, it was inferred that these seven ginkgo chromosomes were ancestral for gymnosperms. The remaining five chromosomes (chr1, 2, 3, 4, and 10) of ginkgo exhibited CRT patterns when compared with conifer genomes, whereas such patterns were absent in *C. panzhihuaensis* (Figure S1, Supporting Information). Despite lacking an outgroup with conserved synteny to determine the ancestral state for a CRT event, the *G. biloba* chromosomes were arbitrarily assigned as ancestral, supported by synteny patterns with *C. panzhihuaensis* (Figure S1A, Supporting Information). Considering that the time span from the MRCA of gymnosperms to the MRCA of the Pinaceae (≈200 Myr) is much greater than that from the MRCA of gymnosperms to the MRCA of *G. biloba* + *C. panzhihuaensis* (≈10–20 Myr),^[^ ^24^, ^45^ ^]^ it is most likely that the CRTs between *G. biloba* and the Pinaceae occurred in the ancestry of the Pinaceae, rather than in the ancestry of *G. biloba* + *C. panzhihuaensis*. In addition, given that centromere positions are conserved among conifer genomes, the centromeres of the ancestral chromosomes were reconstructed using the arms of *P. squamata*, whose karyotype is more conserved and whose assembly quality is higher compared to other conifer genomes (Table S1, Supporting Information). For the proto‐Pinaceae karyotype (PPK), the ancestral states were clearly inferred with the maximum parsimony criterion (Figure 3A; Figure S9, Supporting Information). For the proto‐Cupressaceae karyotype (PCK), a CRT event occurred between *M. glyptostroboides* and *C. japonica* (Figure 3A; Figure S12, Supporting Information), and the ancestral state could not be determined due to a lack of support from outgroups. Therefore, the state of *M. glyptostroboides* was arbitrarily assigned as ancestral, because its phylogenetic position is more basal than that of *C. japoni*ca.^[^ ^24^, ^25^ ^]^ For the proto‐Cupressales and Taxaceae karyotypes, the ancestral states were undeterminable due to likely saturated reshuffles by CRTs. Syntenic blocks between the PGK and extant genomes were identified using the `‐icl` option of WGDI, with orthologous synteny further identified using SOI (parameter “filter ‐c 0.5”).^[^ ^26^ ^]^ The karyotypes of the extant genomes were then mapped to the PGK using the `‐km` option of WGDI with manual refinements, and visualized using SOI and JCVI.^[^ ^81^ ^]^

### Simulation of Successive CRT Model

The simulation was initialized with 12 chromosomes, each consisting of two arms. Then an evolutionary path was stimulated with 1 to 60 successive CRT events. At each step, two chromosomes were randomly sampled, and one arm was randomly selected from each of the two chromosomes. These two arms were then exchanged, resulting in two recombined chromosomes. Back recombination (similar to back mutation) was allowed. The entire simulation was repeated 1000 times.

## Conflict of Interest

The authors declare no conflict of interest.

## Author Contributions

Y.‐P.M., W.Z., X.‐Q.W., W.‐B.S., and R.‐G.Z. conceived and designed the study. R.‐G.Z. collected and analyzed the data. R.‐G.Z., and H.‐Y.S. prepared figures and tables. H.L., H.S., D.‐T.L., H.Y., and K.‐H.J. provided supports in materials and methodology. R.‐G.Z. and W.Z. wrote the manuscript; W.Z., Y.‐P.M., X.‐Q.W., W.‐B.S., H.L., and R.‐G.Z. revised the manuscript; all authors approved the final manuscript.

## Supporting information



Supporting Information

## Data Availability

The files of the reconstructed ancestral karyotypes were deposited in Figshare (https://doi.org/10.6084/m9.figshare.26403970).
