# Supplementary material for: Convergent Patterns of Karyotype Evolution Underlying Karyotype Uniformity in Conifers
Source: Adv Sci (Weinh). 2024 Dec 25;12(7):2411098. doi: 10.1002/advs.202411098 (PMC11831501; doi:10.1002/advs.202411098)
Supplement: Supplementary file 1 — Supporting Information [file ADVS-12-2411098-s001.pdf]

ADVANCED SCIENCE
Open Access

Supporting Information

for *Adv. Sci.*, DOI 10.1002/advs.202411098

Convergent Patterns of Karyotype Evolution Underlying Karyotype Uniformity in Conifers

Ren-Gang Zhang, Hui Liu, Hong-Yun Shang, Heng Shu, De-Tuan Liu, Hao Yang, Kai-Hua Jia, Xiao-Quan Wang, Wei-Bang Sun*, Wei Zhao* and Yongpeng Ma**

Supporting Information

Convergent Patterns of Karyotype Evolution Underlying Karyotype Uniformity in Conifers

Ren-Gang Zhang, Hui Liu, Hong-Yun Shang, Heng Shu, De-Tuan Liu, Hao Yang, Kai-Hua Jia, Xiao-Quan Wang, Wei-Bang Sun*, Wei Zhao*, and Yong-Peng Ma**

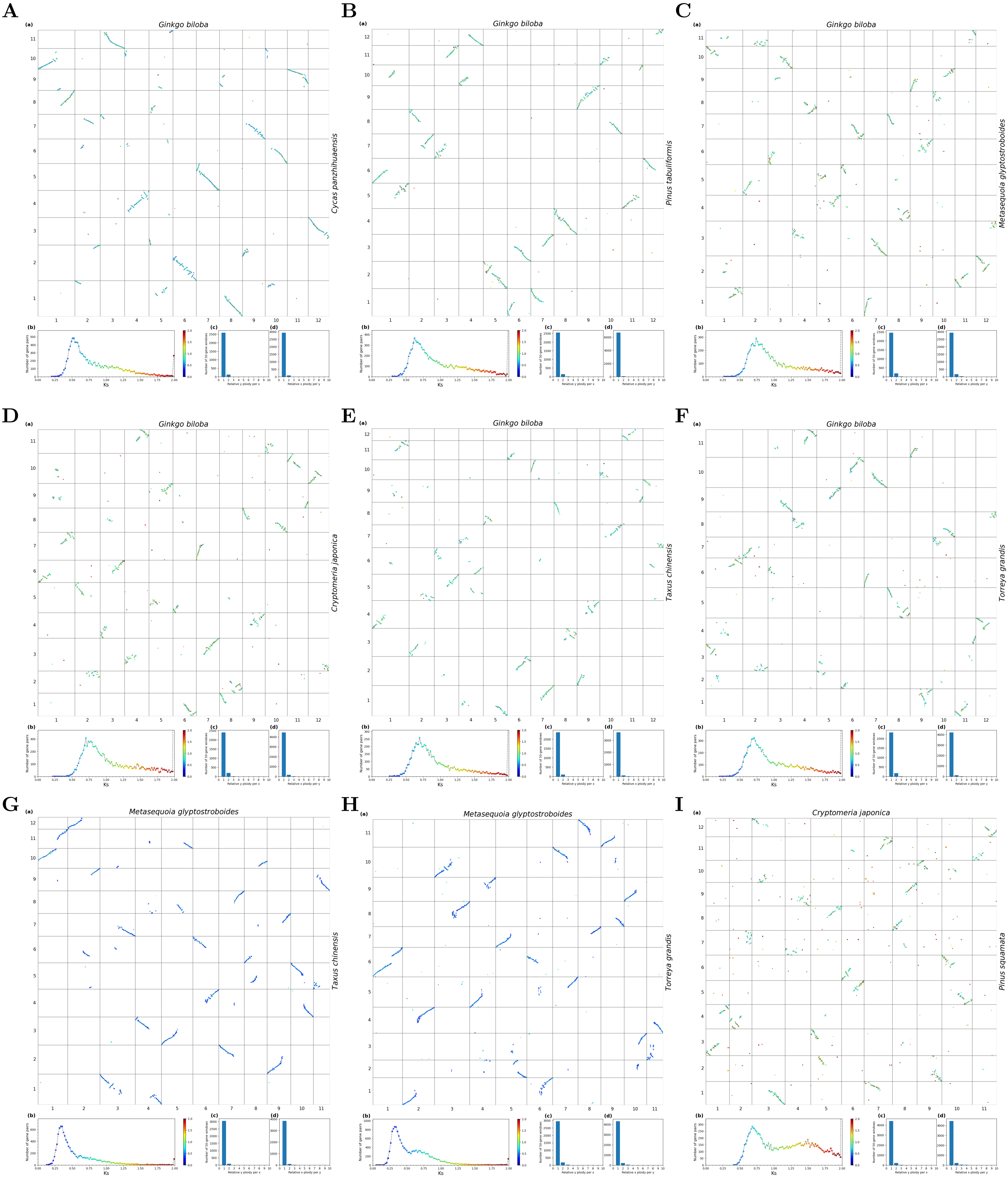

Figure S1. Inter-genomic orthologous synteny between *Ginkgo biloba* and *Cycas panzhihuaensis* (**A**), *Pinus tabuliformis* (**B**), *Metasequoia glyptostroboides* (**C**), *Cryptomeria japonica* (**D**), *Taxus chinensis* (**E**), and *Torreya grandis* (**F**), and between *Metasequoia glyptostroboides* and *Taxus chinensis* (**G**) and *Torreya grandis* (**H**), and between *Cryptomeria japonica* and *Pinus squamata* (**I**), showing clear 1:1 synteny patterns. Other pairwise combinations of these representative species also reveal similar 1:1 synteny patterns (not shown). Subplots: (**a**) dot plots colored by *Ks* to show orthologous gene pairs between two genomes, (**b**) histogram showing the *Ks* distribution of these gene pairs, and (**c–d**) synteny depth (indicative of relative ploidy) across 50-gene windows on the x-axis (c) or y-axis (d) in subplot a.

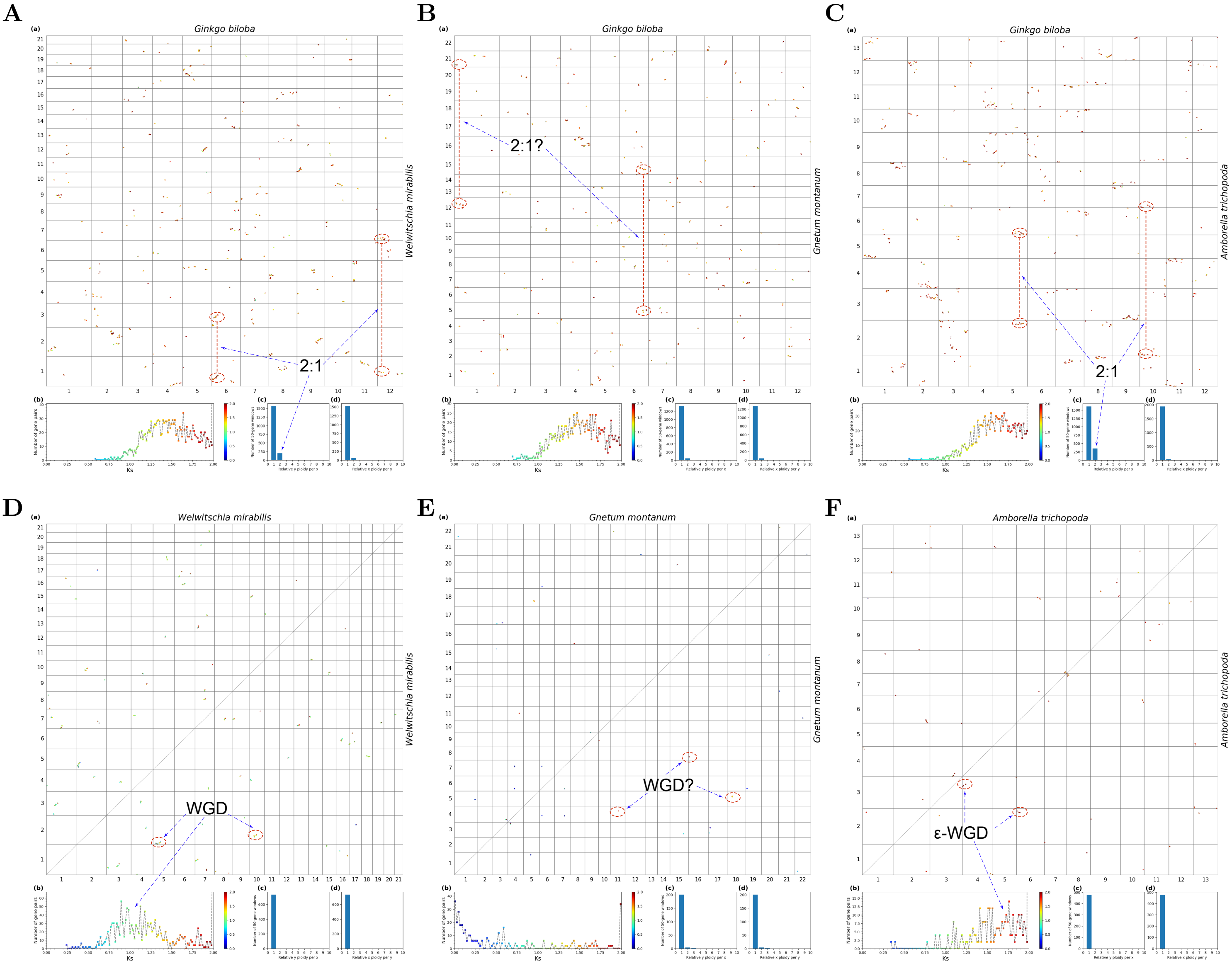

Figure S2. Synteny patterns between *Ginkgo biloba* and *Welwitschia mirabilis* (**A**), *Gnetum montanum* (**B**) and *Amborella trichopoda* (**C**), and within *Welwitschia mirabilis* (**D**), *Gnetum montanum* (**E**) and *Amborella trichopoda* (**F**), showing fragmented syntenic relics of WGD events in lineages of *Welwitschia mirabilis*, *Gnetum montanum* and *Amborella trichopoda*. Subplots: (**a**) dot plots colored by *Ks* to show orthologous or paralogous gene pairs, (**b**) histogram showing the *Ks* distribution of these gene pairs, and (**c–d**) synteny depth (indicative of relative ploidy to a reference) across 50-gene windows on the x-axis (c) or y-axis (d) in subplot a. Examples of synteny patterns are highlighted with dashed ellipses and arrows.

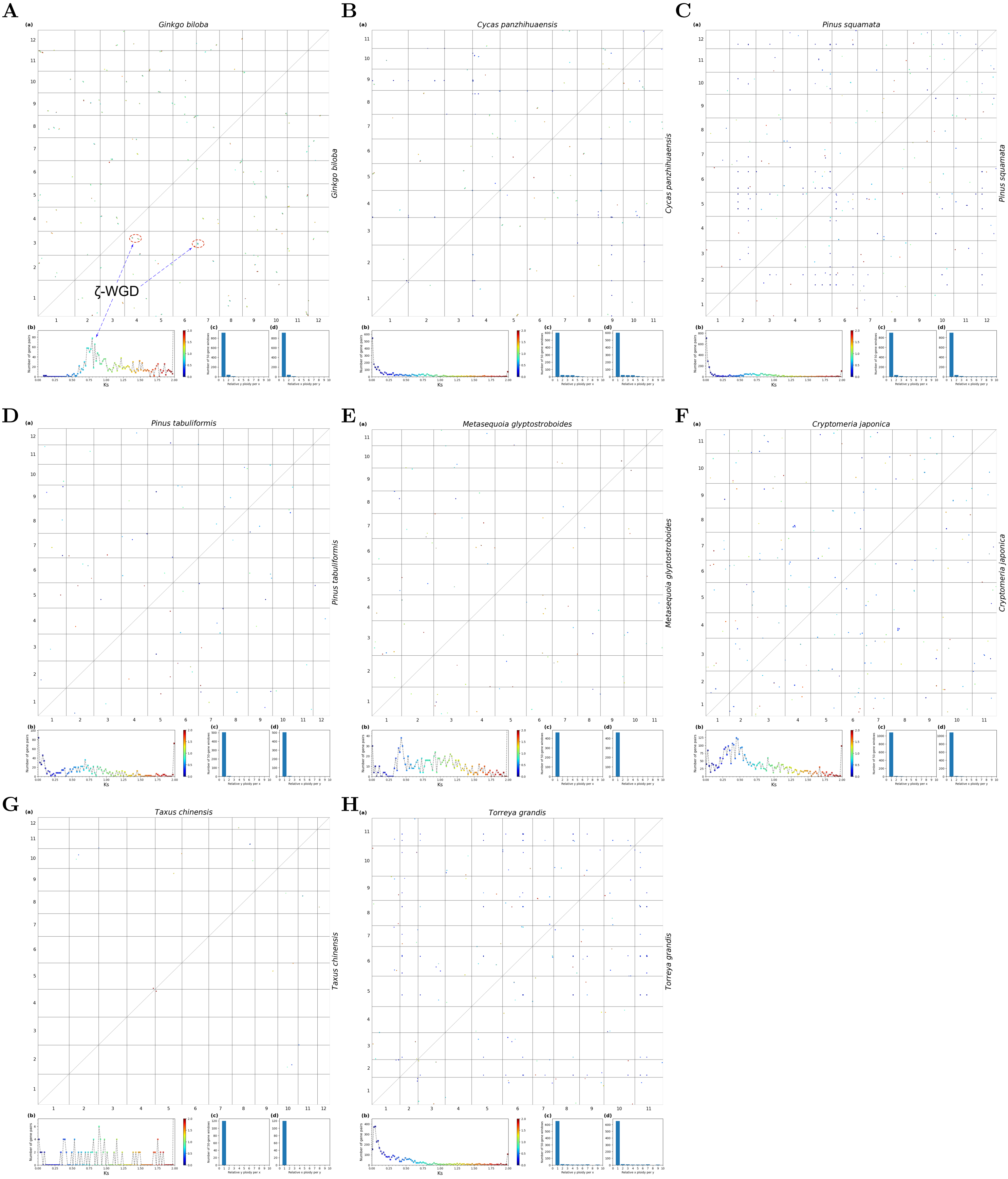

Figure S3. Intra-genomic synteny for *Ginkgo biloba* (**A**), *Cycas panzhihuaensis* (**B**), *Pinus squamata* (**C**), *Pinus tabuliformis* (**D**), *Metasequoia glyptostroboides* (**E**), *Cryptomeria japonica* (**F**), *Taxus chinensis* (**G**) and *Torreya grandis* (**H**). Subplots: (**a**) dot plots colored by *Ks* to show paralogous gene pairs, (**b**) histogram showing the *Ks* distribution of these gene pairs, and (**c–d**) synteny depth across 50-gene windows on the x-axis (c) or y-axis (d) in subplot a. Examples of synteny patterns are highlighted with dashed ellipses and arrows.

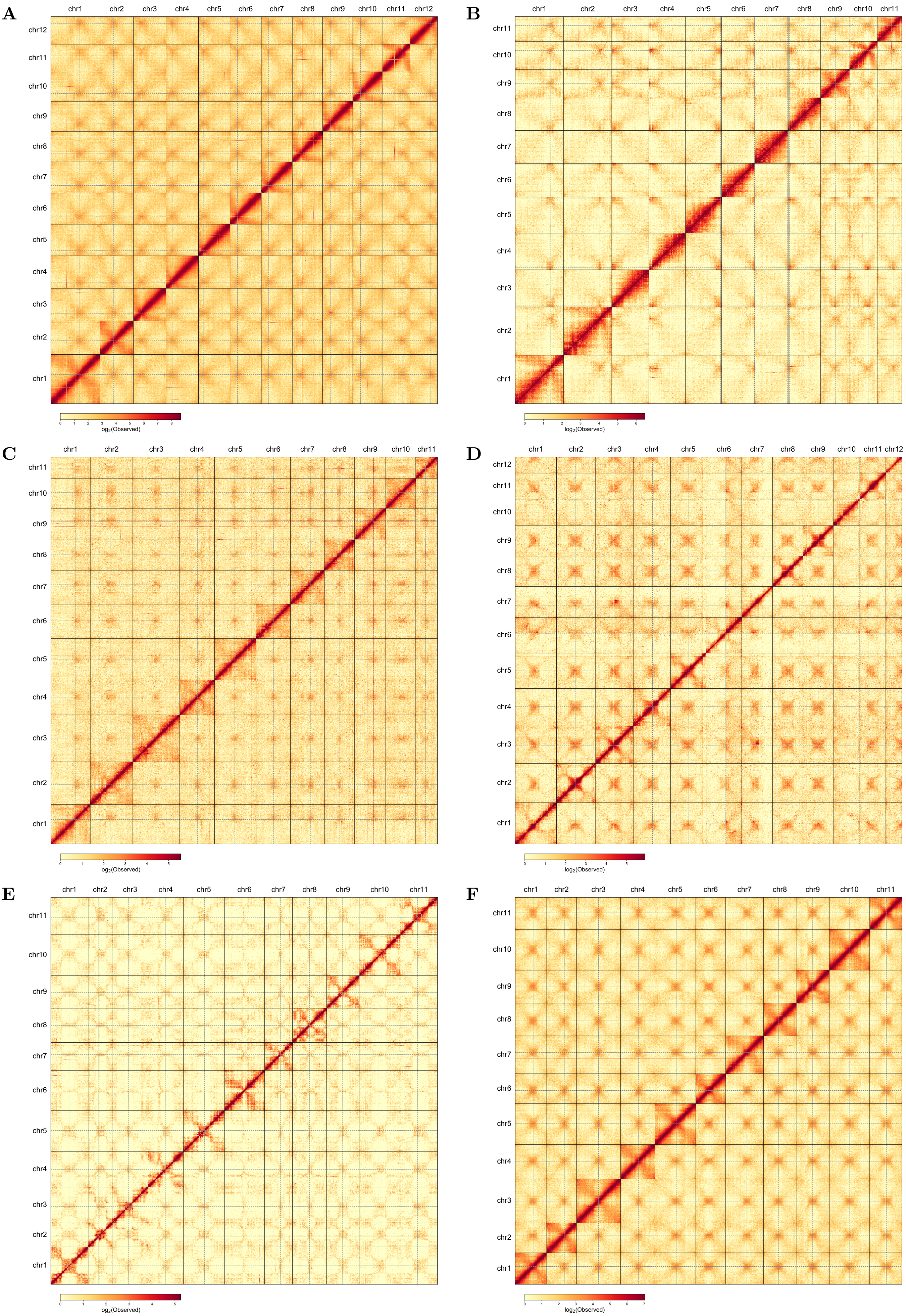

Figure S4. Identification of putative centromere positions for *Ginkgo biloba* (**A**), *Cycas panzhihuaensis* (**B**), *Metasequoia glyptostroboides* (**C**), *Taxus chinensis* (**D**), *Torreya grandis* (**E**) and *Cryptomeria japonica* (**F**), based on the characterized, inter-chromosomal interaction signals between centromere regions in Rabl configurations. The resolution of Hi-C matrices is 1 Mb. Dashed gray lines indicate the centromere positions identified from the Hi-C heatmaps.

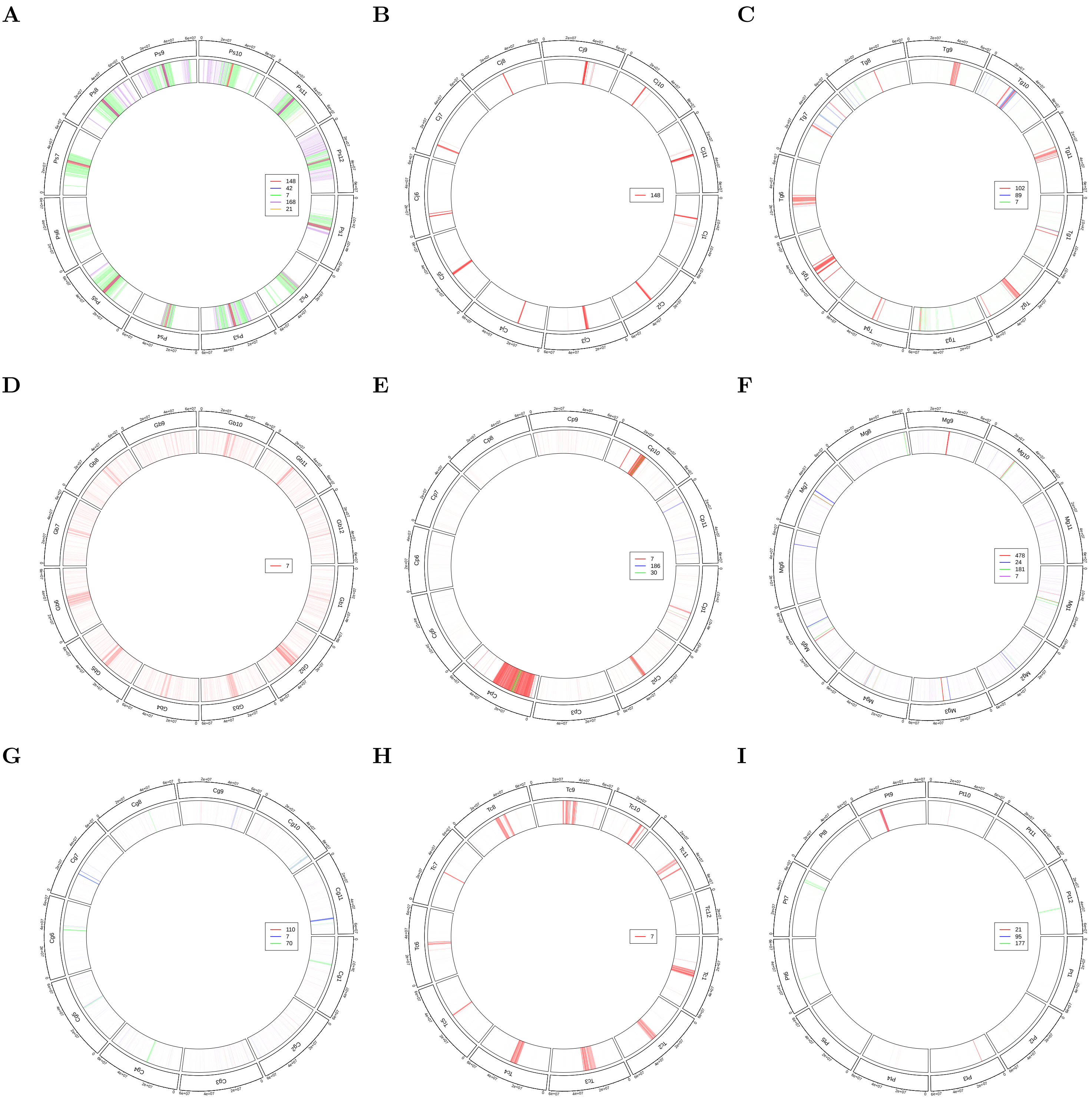

Figure S5. Tandem repeats identified in the pericentromeric regions of *Pinus squamata* (**A**), *Cryptomeria japonica* (**B**), *Torreya grandis* (**C**), *Ginkgo biloba* (**D**), *Cycas panzhihuaensis* (**E**), *Metasequoia glyptostroboides* (**F**), *Cupressus gigantea* (**G**),*Taxus chinensis* (**H**) and *Pinus tabuliformis* (**I**). Circos plots generated by TRASH show the abundant tandem repeats. Colored lines indicate tandem repeats with different motif lengths. The putative centromere positions identified from the Hi-C heatmaps are located at the 30 Mb position of each chromosome.

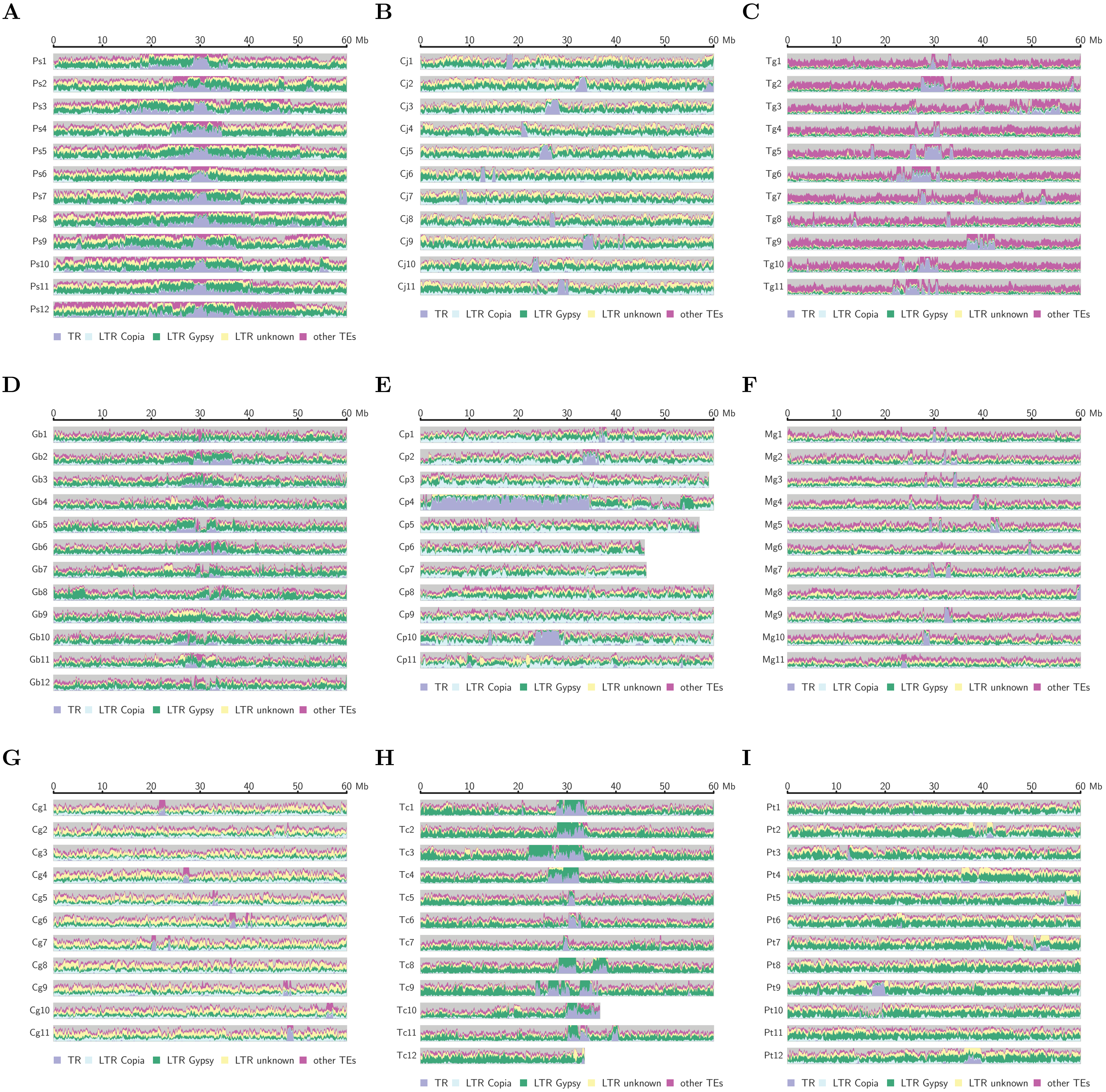

Figure S6. Abundance of tandem repeats and transposable elements in the peri-centromeric regions of *Pinus squamata* (**A**), *Cryptomeria japonica* (**B**), *Torreya grandis* (**C**), *Ginkgo biloba* (**D**), *Cycas panzhihuaensis* (**E**), *Metasequoia glyptostroboides* (**F**), *Cupressus gigantea* (**G**), *Taxus chinensis* (**H**) and *Pinus tabuliformis* (**I**). TR, tandem repeat; LTR, long terminal repeat retrotransposons; TE, transposable element. The putative centromere positions identified from the Hi-C heatmaps are located at the 30 Mb position of each chromosome.

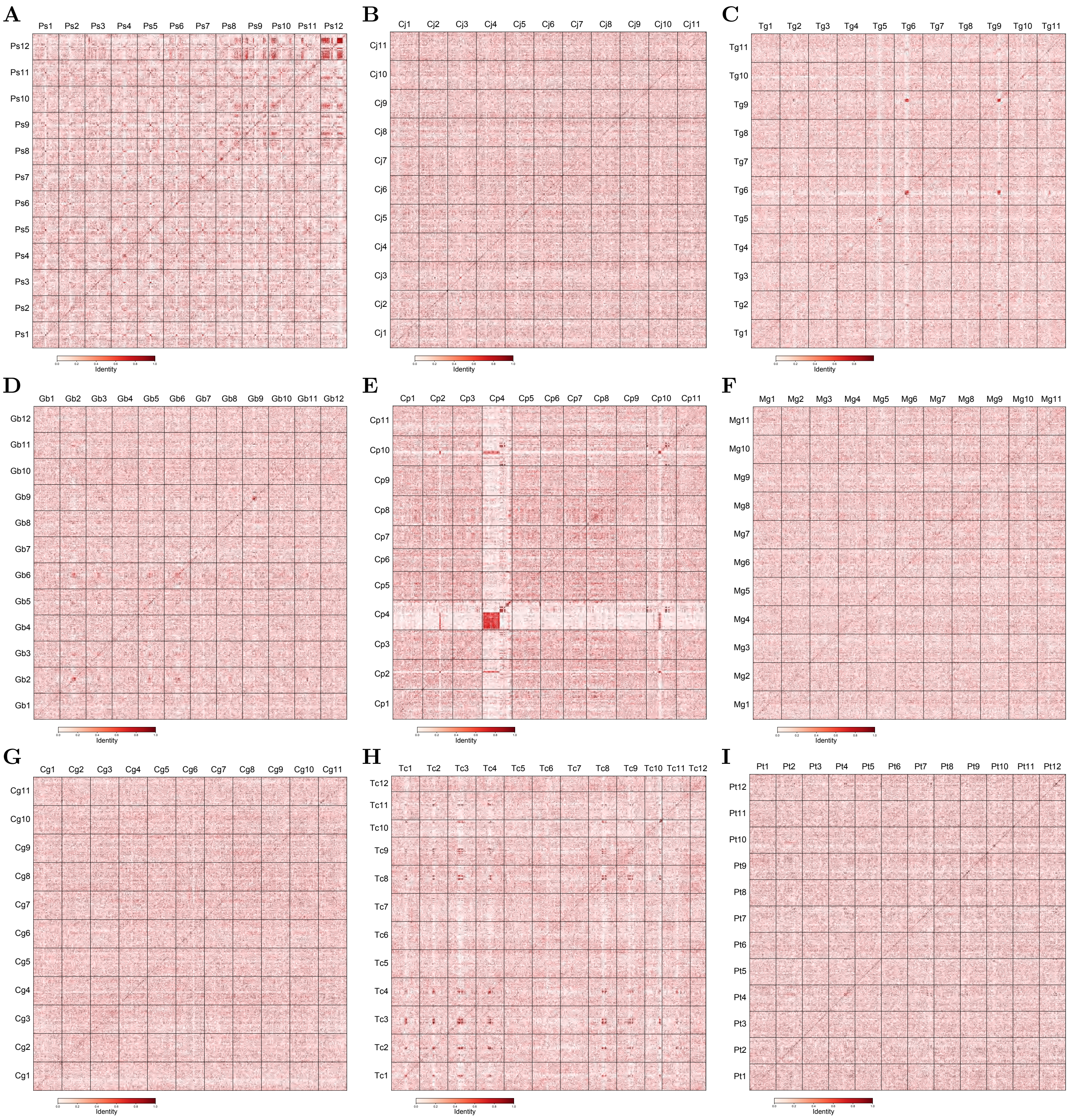

Figure S7. All-versus-all alignments across the pericentromeric regions of *Pinus squamata* (**A**), *Cryptomeria japonica* (**B**), *Torreya grandis* (**C**), *Ginkgo biloba* (**D**), *Cycas panzhihuaensis* (**E**), *Metasequoia glyptostroboides* (**F**), *Cupressus gigantea* (**G**), *Taxus chinensis* (**H**) and *Pinus tabuliformis* (**I**). Red dots represent average sequence identities across 60 kb windows.

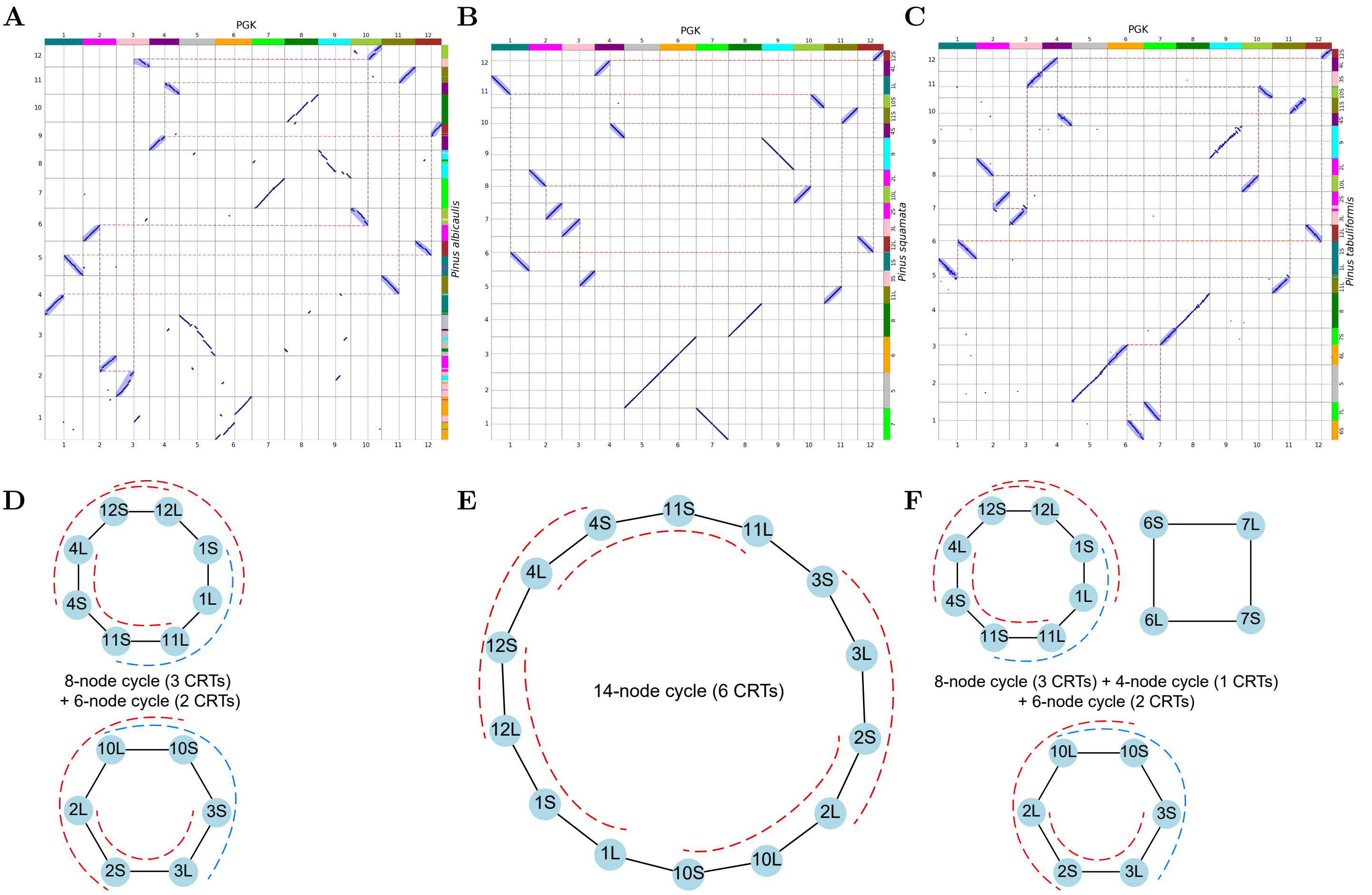

Figure S8. Karyotype evolution of *Pinus* from the PGK. (**A–C**) Dot plots between PGK and *Pinus albicaulis* (A), *Pinus squamata* (B) and *Pinus tabuliformis* (C). Numbers along the bottom and left axes of dot plots represent chromosomes in the PGK and modern genomes, respectively. Colored panels and numbered labels on the top and right axes indicate the mapped chromosomes/arms of the PGK. Dashed gray lines denote centromere positions. Reshuffled arms are highlighted with shadows, and breakpoints are linked by red dashed squares. (**D–F**) Graphs showing arm-reshuffling between PGK and *Pinus albicaulis* (D), *Pinus squamata* (E) and *Pinus tabuliformis* (F). In the graphs, nodes (arm numbers in colored circles) correspond to the arm numbers of the PGK, and edges (black lines) indicate links by breakpoints. The arm identification numbers of the nodes correspond to those along the top and right axes of the dot plots (A–C). Dashed curves indicate shared paths (breakpoints) among the graphs.

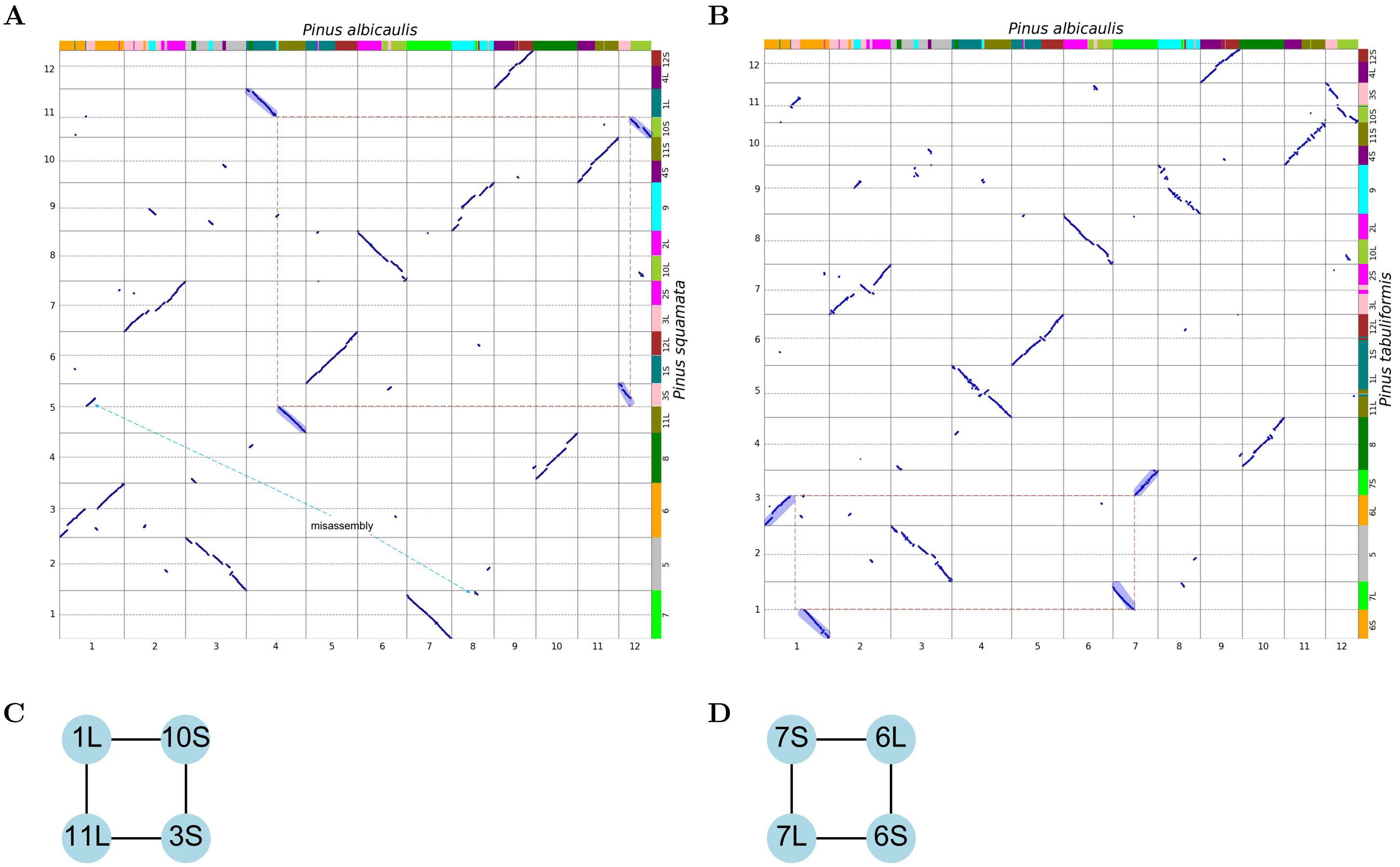

Figure S9. Karyotype evolution within *Pinus*. (**A–B**) Dot plots between *Pinus albicaulis* and *Pinus squamata* (A) and *Pinus tabuliformis* (B). Numbers along the bottom and left axes of dot plots represent chromosomes in the modern genomes. Colored panels and numbered labels on the top and right axes indicate the mapped chromosomes/arms of the PGK. Dashed gray lines denote centromere positions. Reshuffled arms are highlighted with shadows, and breakpoints are linked by red dashed squares. The large-scale misassembly in the *Pinus albicaulis* genome was identified using a Hi-C heatmap (not shown). (**C–D**) Graphs showing arm-reshuffling between *Pinus albicaulis* and *Pinus squamata* (C) and *Pinus tabuliformis* (D). Arm identification numbers of the nodes correspond to those along the right axis of the dot plots (A–B).

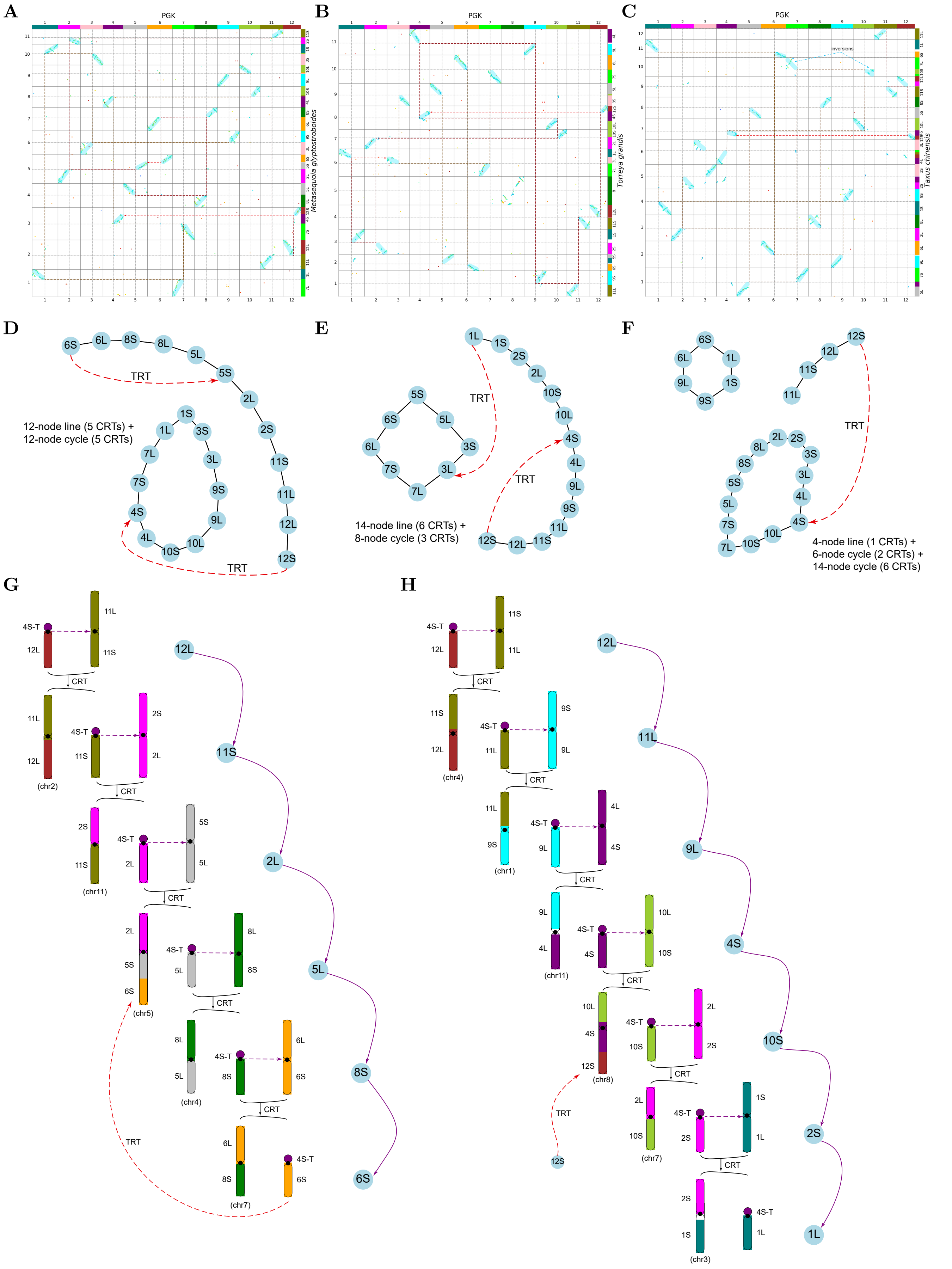

Figure S10. Karyotype evolution of the Cupressales from the PGK. (**A–C**) Dot plots between the PGK and *Metasequoia glyptostroboides* (A), *Torreya grandis* (B), and *Taxus chinensis* (C). Numbers along the bottom and left axes represent chromosomes in the PGK and modern genomes, respectively. Colored panels and numbered labels on the top and right axes indicate the mapped chromosomes/arms of the PGK. Dashed gray lines denote centromere positions. Reshuffled arms are highlighted with shadows. Long segments that are not highlighted are likely due to breakpoints shifting and are ignored. Breakpoints are linked by red dashed polygons or arrows. (**D–F**) Graphs showing arm-reshuffling patterns between the PGK and *Metasequoia glyptostroboides* (D), *Torreya grandis* (E), and *Taxus chinensis* (F). Nodes correspond to the arms of the PGK, and edges (black lines) indicate links by breakpoints. Arm numbers of the nodes correspond to those along the top and right axes of the dot plots (A–C). Red dashed arrows indicate arms joining to the telomeric end of another arm via TRTs. (**G–H**) Schematic diagrams of intermediate CRTs that transfered the arm PGK-4S-T in *Metasequoia glyptostroboides* (G) and *Torreya grandis* (H). Chromosome identification numbers in brackets correspond to those in the extant genomes.

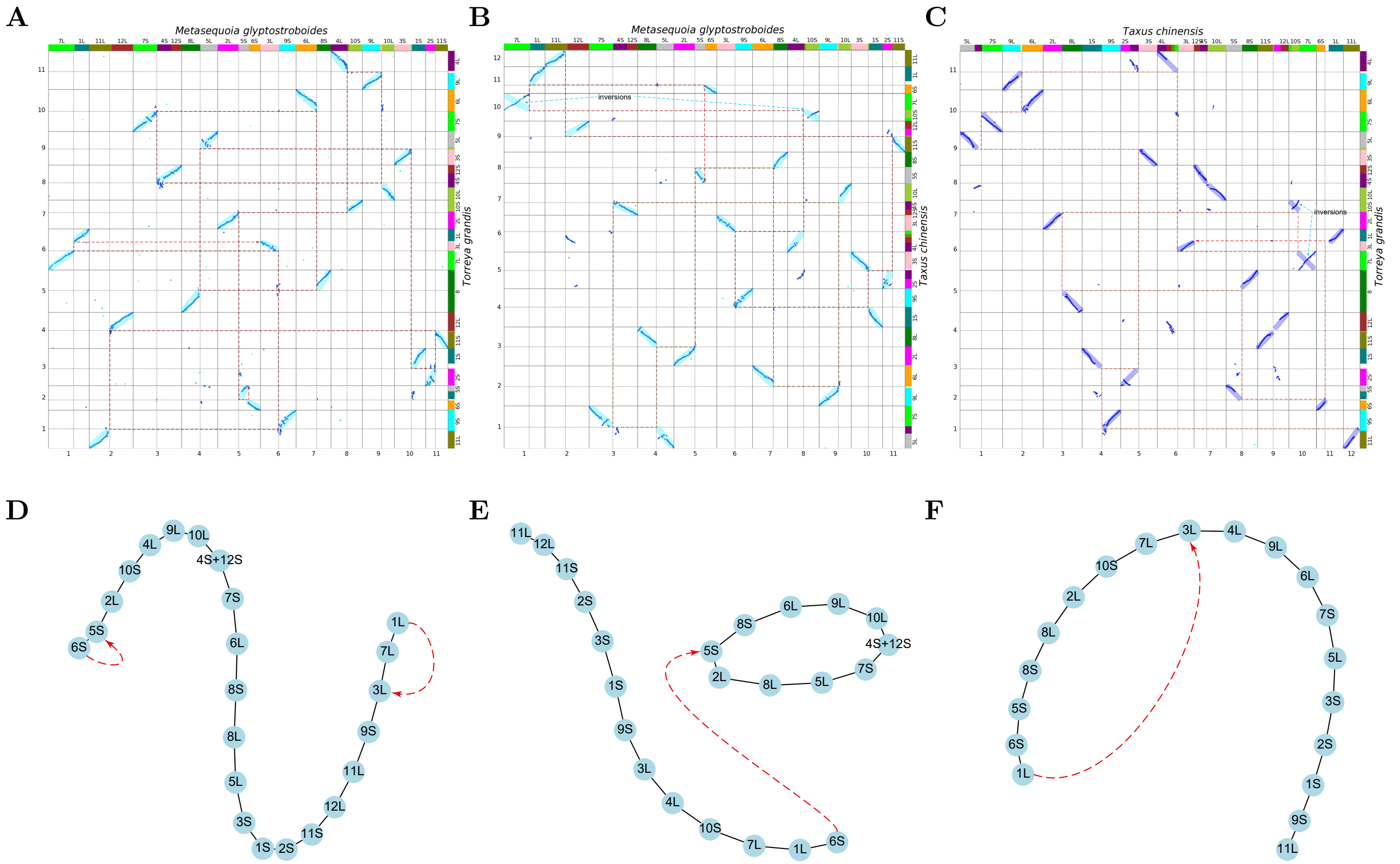

Figure S11. Karyotype evolution within the Cupressales. (**A–C**) Dot plots between *Metasequoia glyptostroboides* and *Torreya grandis* (A) and *Taxus chinensis* (B), and between *Torreya grandis* and *Taxus chinensis* (C). Numbers along the bottom and left axes of the dot plots represent chromosomes in the modern genomes. Colored panels and numbered labels along the top and right axes indicate the mapped chromosomes/arms in the PGK. Dashed gray lines denote centromere positions. Reshuffled arms are highlighted with shadows. Long segments that are not highlighted are likely due to breakpoints shifting and are ignored. Breakpoints are linked by red dashed polygons. (**D–F**) Graphs showing arm-reshuffling between *Metasequoia glyptostroboides* and *Torreya grandis* (D) and *Taxus chinensis* (E), and between *Torreya grandis* and *Taxus chinensis* (F). Nodes correspond to the arms of the PGK, and edges (black lines) indicate links by breakpoints. Arm identification numbers of the nodes correspond to those along the top and right axes of the dot plots (A–C). Red dashed arrows indicate arms joining to the telomeric end of another arm via TRTs.

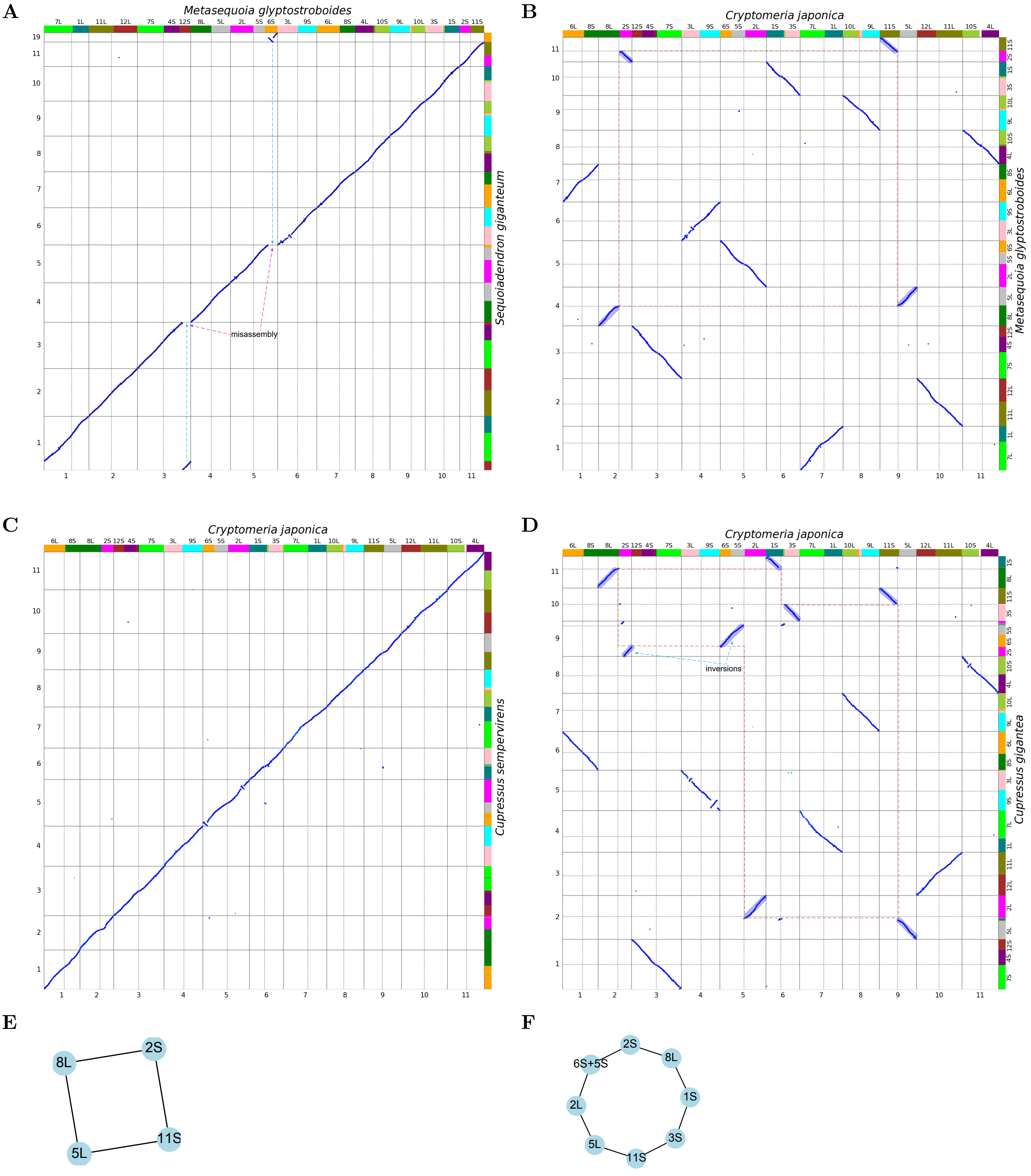

Figure S12. Karyotype evolution within the Cupressaceae. (**A–D**) Dot plots between *Metasequoia glyptostroboides* and *Sequoiadendron giganteum* (A) and *Cryptomeria japonica* (B), and between *Cryptomeria japonica* and *Cupressus sempervirens* (C) and *Cupressus gigantea* (D). Numbers along the bottom and left axes of the dot plots represent chromosomes in the modern genomes. Colored panels and numbered labels along the top and right axes indicate the mapped chromosomes/arms of the PGK. Dashed gray lines denote centromere positions. Reshuffled arms are highlighted with shadows. Breakpoints are linked by red dashed polygons. The large-scale misassembly in the *Sequoiadendron giganteum* genome was identified using a Hi-C heatmap (not shown). (**E–F**) Graphs showing arm-reshuffling between *Metasequoia glyptostroboides* and *Cryptomeria japonica* (E) and between *Cryptomeria japonica* and *Cupressus gigantea* (F). Arm numbers of the nodes correspond to those along the top and right axes of the dot plots (B and D).

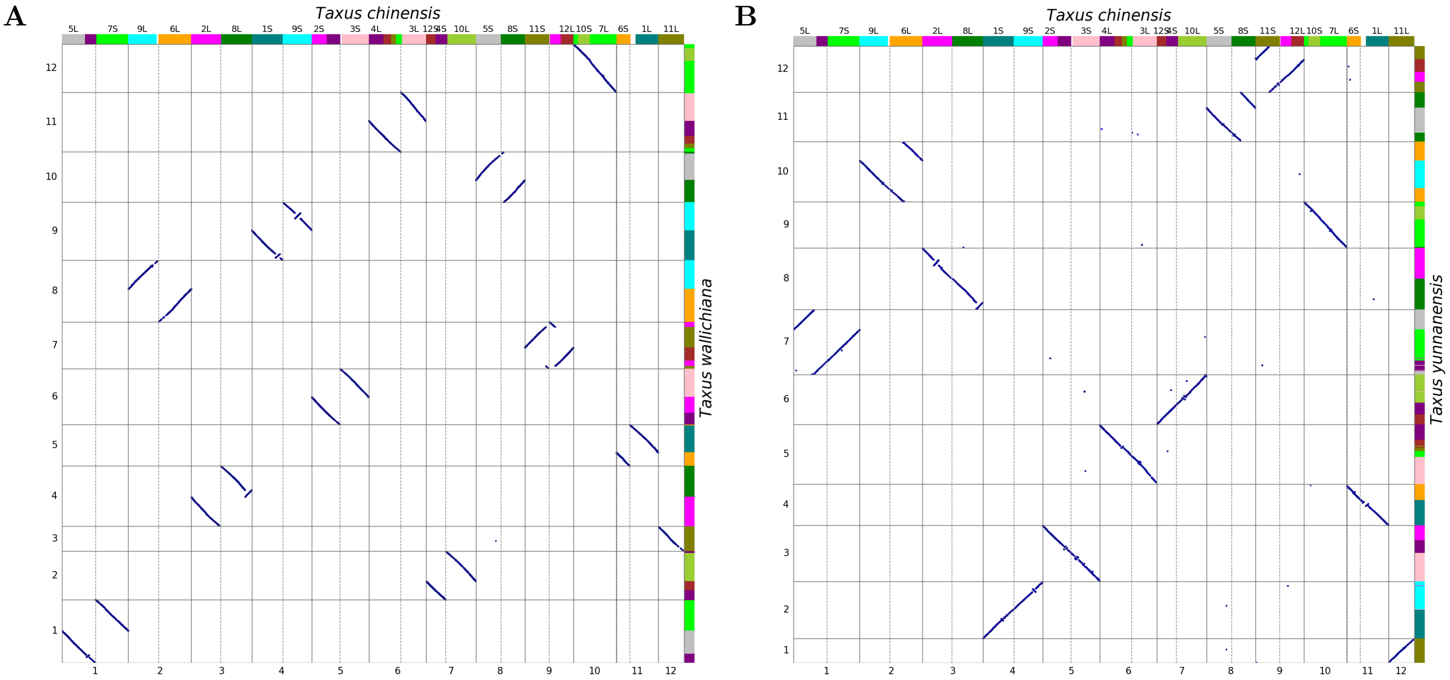

Figure S13. Karyotype evolution within *Taxus*. (**A–B**) Dot plots between *Taxus chinensis* and *Taxus wallichiana* (A) and *Taxus yunnanensis* (B). Numbers along the bottom and left axes of the dot plots represent chromosomes in the modern genomes. Colored panels and numbered labels along the top and right axes indicate the mapped chromosomes/arms of the PGK. Dashed gray lines denote centromere positions. No large-scale inter-chromosomal translocations are observed. The arm-scale inversions in *Taxus wallichiana* (A) and *Taxus yunnanensis* (B) are most likely due to misassembly after reviewing the published Hi-C heatmaps.

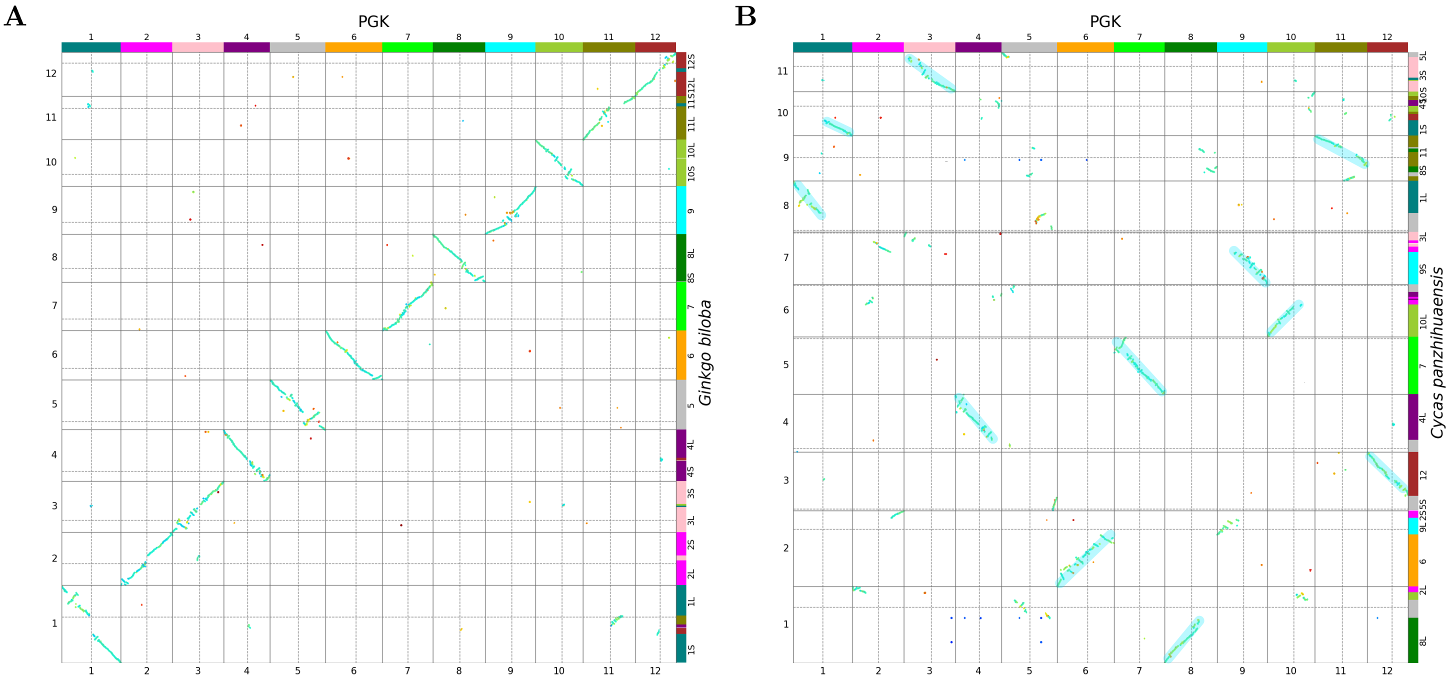

Figure S14. Dot plots between the PGK and *Ginkgo biloba* (**A**) and *Cycas panzhihuaensis* (**B**). Numbers along the bottom and left axes of the dot plots are chromosomes in the PGK and modern genomes, respectively. Colored panels and numbered labels along the top and right axes correspond to the chromosomes/arms of the PGK. Dashed gray lines denote centromere positions. The syntenic majority of each *Cycas panzhihuaensis* chromosomes is highlighted with shadows.

Table S1. Genomes of 16 representative gymnosperms used in this study.

Species	Genome size (bp)	Scaffold N50 (bp)	Contig N50 (bp)	Gene number	Genome BUSCO (n = 1614)	Proteome BUSCO (n = 1614)
Ginkgo biloba	9.88G	754.6M	1.6M	27,836	C:93.43%[S:90.52%,D:2.91%],F:4.09%,M:2.48%	C:63.5%[S:58.2%,D:5.3%],F:19.2%,M:17.3%
Cycas panzhihuaensis	10.48G	952.1M	12.1M	32,353	C:91.26%[S:88.48%,D:2.79%],F:4.65%,M:4.09%	C:90.5%[S:84.6%,D:5.9%],F:2.2%,M:7.3%
Gnetum montanum	4.13G	156.9M	25K	27,354	C:86.80%[S:83.95%,D:2.85%],F:2.23%,M:10.97%	C:83.6%[S:79.2%,D:4.4%],F:4.3%,M:12.1%
Welwitschia mirabilis	6.87G	359.8M	1.5M	26,990	C:82.78%[S:80.92%,D:1.86%],F:6.26%,M:10.97%	C:76.8%[S:73.4%,D:3.4%],F:6.4%,M:16.8%
Pinus tabuliformis	25.42G	2.11G	2.6M	80,495	C:90.95%[S:76.70%,D:14.25%],F:4.71%,M:4.34%	C:84.7%[S:69.0%,D:15.7%],F:6.5%,M:8.8%
Pinus squamata	29.18G	2.48G	915.4M	55,413	C:91.33%[S:87.11%,D:4.21%],F:4.15%,M:4.52%	C:94.9%[S:87.4%,D:7.5%],F:1.5%,M:3.6%
Pinus albicaulis	27.61G	2.01G	537K	25,246	C:84.82%[S:79.37%,D:5.45%],F:8.98%,M:6.20%	C:66.6%[S:62.2%,D:4.4%],F:14.4%,M:19.0%
Sequoiadendron giganteum	8.13G	690.5M	318K	37,936	C:91.76%[S:89.03%,D:2.73%],F:2.79%,M:5.45%	C:50.6%[S:47.3%,D:3.3%],F:14.9%,M:34.5%
Cryptomeria japonica	9.05G	754.8M	8.5M	55,246	C:92.01%[S:89.10%,D:2.91%],F:2.97%,M:5.02%	C:91.6%[S:86.1%,D:5.5%],F:2.6%,M:5.8%
Metasequoia glyptostroboides	8.08G	722.6M	11.8M	32,174	C:91.08%[S:88.85%,D:2.23%],F:2.85%,M:6.07%	C:81.9%[S:77.1%,D:4.8%],F:5.8%,M:12.3%
Cupressus gigantea	10.92G	917.1M	1.6M	35,384	C:91.08%[S:84.94%,D:6.13%],F:3.35%,M:5.58%	C:71.8%[S:66.6%,D:5.2%],F:16.2%,M:12.0%
Cupressus sempervirens	10.01G	919.9M	26.7M	84,157	C:92.19%[S:89.84%,D:2.35%],F:2.60%,M:5.20%	C:86.6%[S:81.6%,D:5.0%],F:5.3%,M:8.1%
Taxus chinensis	10.24G	903.7M	2.4M	44,770	C:88.85%[S:85.19%,D:3.66%],F:4.15%,M:7.00%	C:50.6%[S:46.6%,D:4.0%],F:22.2%,M:27.2%
Taxus yunnanensis	10.74G	966.8M	2.9M	34,931	C:91.64%[S:86.37%,D:5.27%],F:2.85%,M:5.51%	C:52.3%[S:48.6%,D:3.7%],F:16.9%,M:30.8%
Taxus wallichiana	11.12G	987.2M	3.6M	27,764	C:92.13%[S:86.18%,D:5.95%],F:2.73%,M:5.14%	C:88.8%[S:81.1%,D:7.7%],F:2.5%,M:8.7%
Torreya grandis	19.05G	1.82G	2.7M	47,089	C:91.82%[S:85.63%,D:6.20%],F:3.10%,M:5.08%	C:68.0%[S:60.8%,D:7.2%],F:17.2%,M:14.8%

Table S2. Putative centromere positions identified by Hi-C signals in nine representative gymnosperms.

Species	Chromosome	Length (bp)	Centromere position (rough)	Length of long arm	Length of short arm	Arm ratio (long / short arm)	Category*
Metasequoia glyptostroboides	chr1	818,874,304	506,000,000	506,000,000	312,874,304	1.62	m
	chr2	883,923,269	411,000,000	472,923,269	411,000,000	1.15	m
	chr3	973,530,065	477,000,000	496,530,065	477,000,000	1.04	m
	chr4	722,600,678	370,000,000	370,000,000	352,600,678	1.05	m
	chr5	860,062,275	420,000,000	440,062,275	420,000,000	1.05	m
	chr6	715,836,781	366,000,000	366,000,000	349,836,781	1.05	m
	chr7	702,647,723	412,000,000	412,000,000	290,647,723	1.42	m
	chr8	628,347,089	320,000,000	320,000,000	308,347,089	1.04	m
	chr9	646,074,236	386,000,000	386,000,000	260,074,236	1.48	m
	chr10	622,644,211	340,000,000	340,000,000	282,644,211	1.20	m
	chr11	452,563,994	201,000,000	251,563,994	201,000,000	1.25	m
Cryptomeria japonica	chr1	718,876,227	408,000,000	408,000,000	310,876,227	1.31	m
	chr2	680,319,432	402,000,000	402,000,000	278,319,432	1.44	m
	chr3	1,007,622,168	500,000,000	507,622,168	500,000,000	1.02	m
	chr4	781,262,672	403,000,000	403,000,000	378,262,672	1.07	m
	chr5	933,584,362	487,000,000	487,000,000	446,584,362	1.09	m
	chr6	683,545,021	301,000,000	382,545,021	301,000,000	1.27	m
	chr7	862,687,481	483,000,000	483,000,000	379,687,481	1.27	m
	chr8	746,363,822	342,000,000	404,363,822	342,000,000	1.18	m
	chr9	754,841,075	370,000,000	384,841,075	370,000,000	1.04	m
	chr10	924,125,822	457,000,000	467,125,822	457,000,000	1.02	m
	chr11	736,731,119	382,000,000	382,000,000	354,731,119	1.08	m
Taxus chinensis	chr1	1,051,374,208	529,000,000	529,000,000	522,374,208	1.01	m
	chr2	994,982,319	475,000,000	519,982,319	475,000,000	1.09	m
	chr3	957,407,293	465,000,000	492,407,293	465,000,000	1.06	m
	chr4	949,371,534	489,000,000	489,000,000	460,371,534	1.06	m
	chr5	903,737,476	448,000,000	455,737,476	448,000,000	1.02	m
	chr6	908,182,231	502,000,000	502,000,000	406,182,231	1.24	m
	chr7	785,842,148	308,000,000	477,842,148	308,000,000	1.55	m
	chr8	773,273,111	393,000,000	393,000,000	380,273,111	1.03	m
	chr9	769,507,007	383,000,000	386,507,007	383,000,000	1.01	m
	chr10	680,729,269	674,000,000	674,000,000	6,729,269	100.16	t
	chr11	659,955,358	207,000,000	452,955,358	207,000,000	2.19	sm
	chr12	411,570,604	408,000,000	408,000,000	3,570,604	114.27	t
Torreya grandis	chr1	1,824,307,213	935,000,000	935,000,000	889,307,213	1.05	m
	chr2	1,166,843,293	492,000,000	674,843,293	492,000,000	1.37	m
	chr3	1,762,147,815	810,000,000	952,147,815	810,000,000	1.18	m
	chr4	1,717,786,663	860,000,000	860,000,000	857,786,663	1.00	m
	chr5	2,007,191,503	1,040,000,000	1,040,000,000	967,191,503	1.08	m
	chr6	1,957,849,926	910,000,000	1,047,849,926	910,000,000	1.15	m
	chr7	1,367,468,039	780,000,000	780,000,000	587,468,039	1.33	m
	chr8	1,668,700,658	827,000,000	841,700,658	827,000,000	1.02	m
	chr9	1,587,360,643	762,000,000	825,360,643	762,000,000	1.08	m
	chr10	2,000,250,390	967,000,000	1,033,250,390	967,000,000	1.07	m
	chr11	1,826,763,786	847,000,000	979,763,786	847,000,000	1.16	m
Cycas panzhihuaensis	chr1	1,275,696,759	935,000,000	935,000,000	340,696,759	2.74	sm
	chr2	1,267,120,990	955,000,000	955,000,000	312,120,990	3.06	st
	chr3	979,843,051	29,000,000	950,843,051	29,000,000	32.79	t
	chr4	962,719,060	55,000,000	907,719,060	55,000,000	16.50	t
	chr5	952,059,863	925,000,000	925,000,000	27,059,863	34.18	t
	chr6	880,835,044	865,000,000	865,000,000	15,835,044	54.63	t
	chr7	871,211,421	855,000,000	855,000,000	16,211,421	52.74	t
	chr8	855,569,528	35,000,000	820,569,528	35,000,000	23.44	t
	chr9	750,082,928	385,000,000	385,000,000	365,082,928	1.05	m
	chr10	738,491,204	499,000,000	499,000,000	239,491,204	2.08	sm
	chr11	653,533,439	420,000,000	420,000,000	233,533,439	1.80	sm
Ginkgo biloba	chr1	1,185,857,400	700,000,000	700,000,000	485,857,400	1.44	m
	chr2	809,344,006	331,000,000	478,344,006	331,000,000	1.45	m

Species	Chromosome	Length (bp)	Centromere position (rough)	Length of long arm	Length of short arm	Arm ratio (long / short arm)	Category*
	chr3	783,846,588	183,000,000	600,846,588	183,000,000	3.28	st
	chr4	783,425,633	148,000,000	635,425,633	148,000,000	4.29	st
	chr5	762,930,734	128,000,000	634,930,734	128,000,000	4.96	st
	chr6	754,572,270	178,000,000	576,572,270	178,000,000	3.24	st
	chr7	747,809,235	178,000,000	569,809,235	178,000,000	3.20	st
	chr8	731,980,586	212,000,000	519,980,586	212,000,000	2.45	sm
	chr9	728,898,801	185,000,000	543,898,801	185,000,000	2.94	sm
	chr10	708,905,408	188,000,000	520,905,408	188,000,000	2.77	sm
	chr11	669,792,622	484,000,000	484,000,000	185,792,622	2.61	sm
	chr12	667,571,075	505,000,000	505,000,000	162,571,075	3.11	st
Pinus squamata	chr01	2,393,509,474	1,190,500,000	1,203,009,474	1,190,500,000	1.01	m
	chr02	2,630,835,269	1,332,000,000	1,332,000,000	1,298,835,269	1.03	m
	chr03	2,704,521,248	1,439,500,000	1,439,500,000	1,265,021,248	1.15	m
	chr04	2,484,805,029	1,278,500,000	1,278,500,000	1,206,305,029	1.06	m
	chr05	2,446,417,745	1,311,000,000	1,311,000,000	1,135,417,745	1.17	m
	chr06	2,574,235,822	1,401,500,000	1,401,500,000	1,172,735,822	1.20	m
	chr07	2,505,053,227	1,300,000,000	1,300,000,000	1,205,053,227	1.09	m
	chr08	2,491,148,637	1,254,000,000	1,254,000,000	1,237,148,637	1.03	m
	chr09	2,396,012,812	1,122,500,000	1,273,512,812	1,122,500,000	1.12	m
	chr10	2,246,637,353	1,070,000,000	1,176,637,353	1,070,000,000	1.09	m
	chr11	2,406,395,776	993,000,000	1,413,395,776	993,000,000	1.41	m
	chr12	1,893,439,257	1,129,500,000	1,129,500,000	763,939,257	1.49	m
Pinus tabuliformis	chr1	2,364,278,061	1,221,482,357	1,221,482,357	1,142,795,704	1.07	m
	chr2	2,317,450,362	1,131,872,917	1,185,577,445	1,131,872,917	1.05	m
	chr3	2,291,775,479	1,247,369,644	1,247,369,644	1,044,405,835	1.19	m
	chr4	2,192,534,405	1,081,334,100	1,111,200,305	1,081,334,100	1.03	m
	chr5	2,148,190,925	991,165,173	1,157,025,752	991,165,173	1.17	m
	chr6	2,107,674,557	1,133,003,227	1,133,003,227	974,671,330	1.16	m
	chr7	2,082,167,746	1,002,807,761	1,079,359,985	1,002,807,761	1.08	m
	chr8	2,081,484,518	974,253,054	1,107,231,464	974,253,054	1.14	m
	chr9	2,024,734,096	1,071,935,802	1,071,935,802	952,798,294	1.13	m
	chr10	1,752,849,333	782,922,125	969,927,208	782,922,125	1.24	m
	chr11	1,650,012,615	702,348,255	947,664,360	702,348,255	1.35	m
	chr12	1,392,452,741	798,741,524	798,741,524	593,711,217	1.35	m
Cupressus gigantea	chr1	1,189,333,177	593,438,924	595,894,253	593,438,924	1.00	m
	chr2	1,038,383,141	538,774,892	538,774,892	499,608,249	1.08	m
	chr3	1,022,110,063	467,834,839	554,275,224	467,834,839	1.18	m
	chr4	984,861,827	366,496,447	618,365,380	366,496,447	1.69	sm
	chr5	964,166,738	460,772,690	503,394,048	460,772,690	1.09	m
	chr6	917,075,060	417,852,248	499,222,812	417,852,248	1.19	m
	chr7	901,250,477	495,748,500	495,748,500	405,501,977	1.22	m
	chr8	875,175,489	413,225,411	461,950,078	413,225,411	1.12	m
	chr9	830,486,900	719,390,484	719,390,484	111,096,416	6.48	st
	chr10	790,295,524	400,390,665	400,390,665	389,904,859	1.03	m
	chr11	747,295,617	447,332,887	447,332,887	299,962,730	1.49	m

*Category (arm ratio in square brackets): m, metacentric [1.00–1.67]; sm, sub-metacentric [1.68–3.00]; st, sub-telocentric [3.01–7.00]; t, telocentric [> 7.00].
